# Depletion Flocculation of High Internal Phase Pickering Emulsion Inks: A Colloidal Engineering Approach to Develop 3D Printed Porous Scaffolds with Tunable Bioactive Delivery

**DOI:** 10.1021/acsami.4c11035

**Published:** 2024-08-07

**Authors:** Mahdiyar Shahbazi, Henry Jäger, Delphine Huc-Mathis, Peyman Asghartabar Kashi, Rammile Ettelaie, Anwesha Sarkar, Jianshe Chen

**Affiliations:** † Institute of Food Technology, University of Natural Resources and Life Sciences (BOKU), Muthgasse 18, 1190 Vienna, Austria; ‡ Université Paris-Saclay, INRAE, AgroParisTech, UMR SayFood, 91300 Massy, France; § Faculty of Biosystem, College of Agricultural and Natural Resources, Tehran University, 31587-77871 Karaj, Iran; ∥ Food Colloids and Bioprocessing Group, School of Food Science and Nutrition, University of Leeds, Leeds LS2 9JT, U.K.; ⊥ Food Oral Processing Laboratory, School of Food Science & Biotechnology, Zhejiang Gongshang University, Hangzhou 310018, China

**Keywords:** Pickering emulsion, bridging flocculation, depletion stabilization, nonlinear rheology, 3D printing, drug release

## Abstract

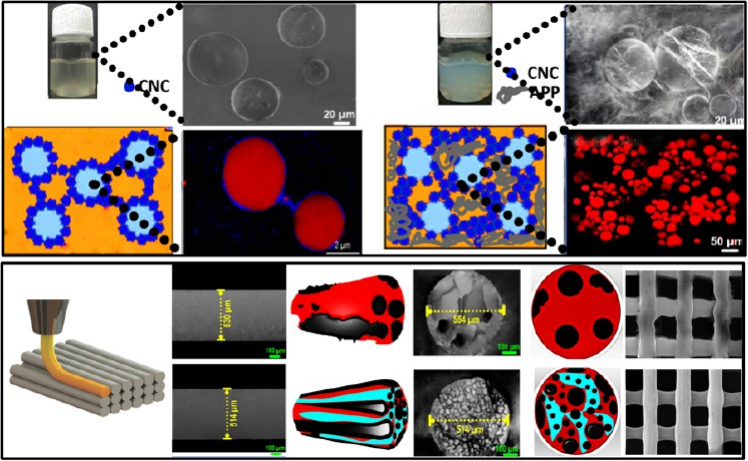

Flocculation is a type of aggregation where the surfaces of approaching droplets are still at distances no closer than a few nanometers while still remaining in close proximity. In a high internal-phase oil-in-water (O/W) emulsion, the state of flocculation affects the bulk flow behavior and viscoelasticity, which can consequently control the three-dimensional (3D)-printing process and printing performance. Herein, we present the assembly of O/W Pickering high-internal-phase emulsions (Pickering-HIPEs) as printing inks and demonstrate how depletion flocculation in such Pickering-HIPE inks can be used as a facile colloidal engineering approach to tailor a porous 3D structure suitable for drug delivery. Pickering-HIPEs were prepared using different levels of cellulose nanocrystals (CNCs), co-stabilized using “raw” submicrometer-sized sustainable particles from a biomass-processing byproduct. In the presence of this sustainable particle, the higher CNC contents facilitated particle-induced depletion flocculation, which led to the formation of a mechanically robust gel-like ink system. Nonetheless, the presence of adsorbed particles on the surface of droplets ensured their stability against coalescence, even in such a highly aggregated system. The gel structures resulting from the depletion phenomenon enabled the creation of high-performance printed objects with tunable porosity, which can be precisely controlled at two distinct levels: first, by introducing voids within the internal structure of filaments, and second, by generating cavities (pore structures) through the elimination of the water phase. In addition to printing efficacy, the HIPEs could be applied for curcumin delivery, and *in vitro* release kinetics demonstrated that the porous 3D scaffolds engineered for the first time using depletion-flocculated HIPE inks played an important role in 3D scaffold disintegration and curcumin release. Thus, this study offers a unique colloidal engineering approach of using depletion flocculation to template 3D printing of sustainable inks to generate next-generation porous scaffolds for personalized drug deliveries.

## 1 Introduction

Three-dimensional (3D) printing has recently emerged as an innovative technology for advanced manufacturing of materials.^1−3^ To accomplish the 3D-printing process to develop custom-designed functional 3D structures, there is a necessity to create a fundamental understanding of ink-based materials and the factors affecting their rheological properties concerning printing performance.^4^ The rheological behaviors of printing inks strongly affect their printability and printing precision, determining their suitability to enhance the multifunctional properties of 3D end-products.^5^ A well-defined printable ink for an effective printing process should possess excellent *pseudoplasticity* as it should easily extrude out through the nozzle tip. Moreover, a viscoelastic ink with enhanced mechanical strength with appropriate thixotropic properties is also crucial to support the designed structure and preserve 3D-printed architectures after the printing process.^6^ The printing ink based on colloidal emulsion systems has been a preferred choice to prepare a *shear-thinning*, viscoelastic, and thixotropic precursor to engineer advanced 3D structures.^7^ As a functionalized printing ink with excellent colloidal stability against coalescence, Pickering emulsions have recently attracted significant interest in 3D-printing applications, as they can endure repeated mechanical deformation and rising temperature during the direct-ink-write (DIW) printing process, attaining improved printability and shape fidelity.^8^

Although stable to coalescence and Ostwald ripening, Pickering emulsion systems are prone to flocculation, which is a universal phenomenon affecting the physical stability, flow behavior, and structure of such emulsions.^9^ Often the dispersed droplets in the O/W emulsions are present in the form of small/large clusters (“flocs”), more prevalent in Pickering emulsions that contain a high-volume fraction of the dispersed phase, also known as Pickering-high-internal-phase emulsions (Pickering-HIPEs).

Flocculation can arise for a variety of reasons, but two of the most commonly encountered forms are bridging and depletion flocculation.^10^ The flocculation can be further influenced by the characteristics of the particles at the O/W interface in addition to the concentration of the particles. One beneficial but rather underestimated significance of this flocculation phenomenon is the formation of a network of droplets, providing a gel-like architecture. Unlike true-Pickering effects, this can not only enhance the stability of the droplets through bulk networking effects jamming the droplets,^11^ but also can be advantageous in 3D printing by providing a reliable method for printing materials without the need for additional rheology modifiers. Provided a high concentration of particles is present in the emulsion, the networks of aggregated particles could form a filled structure throughout the system, encompassing the dispersed droplets as active fillers.^11,12^ As an alternative, introducing non-adsorbing well-dispersed particles to the emulsion can also have an impact on this network creation. A given level of added particles may induce colloidal flocculation via a depletion mechanism. Put simply, the depletion of particles from the gaps between the droplets leads to an osmotic pressure difference between the gap between the particle-laden droplet and the continuous phase, thus pushing the droplets together. At a maximum level of added particles, the Pickering emulsion converts to a colloidally stable system, which is associated with depletion stabilization largely driven by the network in addition to interfacial stabilization.

In 3D printing of emulsion systems, the development of an aggregated network of droplets is of prime importance.^13,14^ It is important to arrange for a suitable level of flocculation in the system to form an emulsion gel but without coalescence of droplets. For surfactant-stabilized (and even polymeric) emulsions, flocculation is almost always preceded by coalescence. However, the exceptional stability of Pickering emulsions against coalescence is what makes them particularly attractive for the current purpose of realizing 3D-printing inks. Efficient controlling of depletion flocculation can be an excellent rheology modification strategy that is principally unexplored in the 3D-printing process, yet can be beneficial to ensure the reliable production of 3D structures concerning printability, printing accuracy, and shape retention.^13^ Altogether, emulsions with depletion flocculation tend to show higher viscosity, more shear-thinning behavior, and greater viscoelasticity than very-well-dispersed individual droplets. The full strength of *pseudoplastic* and viscoelastic inks is strongly associated with the formation of an aggregated droplet network. This type of shear thinning, which starts from moderately low shear values, in emulsion systems is a sure sign of the presence of large, ramified flocs of aggregated droplets. This increasingly breaks into smaller clusters under the influence of applied shear, leading to observed shear-thinning behavior. This is the actual explanation for a *pseudoplastic* printable ink, which can effortlessly squeeze out through the small nozzle tip, while immediately postprinting assures the maintenance of the printed shape owing to the depletion-flocculation-induced gel network architecture. Of more importance, a significant yield stress arises because the large network of aggregated emulsion droplets is high enough to survive the stresses applied to it up to a certain point. This is again important for the printed structures to maintain their own weight once printed. Without a suitable droplet network, the viscosity can be very low as well as Newtonian-like, thus making the formulation unsuitable as a 3D ink.^14^

Advanced structures with intricate porosity are created through a novel process that combines 3D printing with emulsion templating, allowing for precise deposition and formation of complex structures in three-dimensional space. Especially, Pickering-HIPEs have come to the forefront as pioneering printing precursors to foster the effectual engineering of macroporous objects.^15−23^ Classically, Pickering-HIPEs are stabilized by colloidal particles offered by inorganic particles,^24^ synthetic polymers,^25,26^ and green natural ingredients.^27^ Among the different sustainable particles, the current status of nanocelluloses arises partly from their rod-like cellulose nanocrystal (CNC), tailorable crystallinity, nanosized lateral dimension, and structural abilities, equipping them for numerous applications.^28^ In this sense, the irreversibly adsorb at the oil–water interfaces in the O/W emulsions and typically prevent droplet coalescence in such systems. This makes them suitable for preparing printable inks for 3D-printing purposes. irreversibly adsorb at the oil–water interfaces in the O/W emulsions and typically induce colloidal stability against droplet coalescence in such systems. This makes them suitable for preparing printable inks for 3D-printing purposes.

Often, co-stabilizers such as other proteins or sustainable particles of cellulose nanomaterials bring synergism to prepare stable emulsions without hydrophobic modification,^29^ which can tailor a macroporous structure by changing the relative level of CNC.^30^ Therefore, the application of co-stabilizers can also be an effective approach to preparing green particle-stabilized O/W Pickering-HIPEs and promoting depletion flocculation by modulating the concentration of the co-stabilizers. Recently, there has been huge attention to preparing Pickering emulsions from natural organic particles other than starch- and cellulose-based particles, especially raw micron-sized particles obtained from nonpurified (whole) biomass-processing byproducts.^31−33^ The presence of nonsoluble particles (cellulose, lignin, hemicellulose, etc.) and soluble compounds (protein, pectin, etc.) seems to play an important role in the emulsion stability against coalescence, with regard to complementary features at the O/W interface, as well as in the aqueous continuous phase acting to provide the necessary depletion flocculation.

Herein, we demonstrate how a macroporous 3D-printed structure can be designed and material properties can be controlled benefiting from a “depletion floc” Pickering-HIPE-based ink stabilized by a hybrid of CNC and a sustainable biomass-processing byproduct, *i.e.*, apple pomace particles (APP). We hypothesized that the integration of depletion-flocculated Pickering-HIPEs in 3D printing offers the production of macroporous structures with an improved bioactive delivery ability. Accordingly, the HIPE-based inks were stabilized by either CNC or a hybrid of CNC and APP, which could be utilized as a carrier for a bioactive hydrophobic compound; as well as the design of a custom-designed scaffold for potential biofabrication. The preparation conditions to fabricate a 3D macroporous structure were investigated by evaluation of rheological features, morphological characterization, and colloidal stability of Pickering-HIPEs. Strikingly, our findings demonstrate that the microscopic pore structure and mechanical strength of 3D-printed Pickering-HIPEs were improved in addition to quantitative printing time, printing performance, and shape fidelity by depletion flocculation. The resulting mechanically robust porous structure exhibits a hierarchical porous architecture, characterized by multiple opening diameters in the nanometer range. This unique feature enables facile water diffusion through the matrix, thereby inducing the release of curcumin (a model drug) and subsequent disintegration of the scaffold.

## 2 Methods and Materials

### 2.1 Materials

Raw apple pomace powder (APP) (water content: 11.5 wt %/wt, Brix degree: 5.1°, protein content: 6.4 wt %/wt, starch content: 1.0 wt %/wt) was provided from Haisheng Fresh Fruit Juice Co. (Xi’an, Shaanxi, China). Cellulose nanocrystal (CNC) powder was supplied by Qihong Technology Co., Ltd. (Guilin, China). Ultrarefined dewaxed sunflower oil (>98%, Vandermoortele NV, Breda, The Netherlands) possessed a dielectric constant of 2.9 and 98% triglycerides mean values of 70.2 and 25.1% for linoleic and oleic acids, respectively. Commercial phosphate buffer saline (PBS, pH = 7.4) and curcumin from *Curcuma longa* (Turmeric, >65%) were acquired from Sigma-Aldrich (Stuttgart, Germany). The solvent used was deionized water, which is generated by reverse osmosis (Ultra-Purified Type I, 18.2 Megohm water). Other reagents were commercial grade and used without further purification.

### 2.2 Preparation of Pickering-HIPE-Based Inks

To prepare the Pickering-HIPEs, CNC was suspended in distilled water and stirred overnight to ensure complete hydration. All of the Pickering-HIPEs stabilized by CNC or a hybrid of CNC/APP were obtained by homogenizing the mixtures of corn oil 75% w/w oil with 25% w/w aqueous dispersion-contained CNC or CNC/APP suspensions using a high rotor-stator device (SilentCrusher 130 M, Heidolph, Germany) operating at 12,000 min^–1^ for 120 s followed by ultrasound treatments for 600 s.

Specifically, to stabilize the O/W HIPEs, we utilized CNC and APP in two distinct ways to induce the required flocculation of the HIPEs. Four different concentrations of CNC (ranging from 0.2 to 0.8 w/w) with no added APP were utilized to evaluate the ability of CNC to stabilize the O/W HIPEs. The O/W HIPEs, which included CNC, were initially emulsified using a high-shear rotor-stator device (SilentCrusher 130 M, Heidolph, Germany) for 120 s at 12,000 rpm. This was followed by a high-intensity sonication process (VC 750, Sonics & Materials, Inc., CT) with a 13 mm diameter probe for 300 s. The high-intensity emulsification process was performed at a frequency of 20 kHz, with an amplitude of 60% and a power of 450 W, involving a 10 s/4 s on/off cycle.^15,16^ The resulting CNC-containing Pickering-HIPEs have been labeled as E-CNC1, E-CNC2, E-CNC3, and E-CNC4 corresponding to the emulsions containing 0.2, 0.4, 0.6, and 0.8 w/w CNC.

Following this step, a fixed amount of 1.5 w/w APP was added to CNC-containing Pickering-HIPEs, which was optimized based on the physical stability measurement (Supporting Information, Section S1). The resulting CNC-containing Pickering-HIPEs with APP were labeled as E-CNC1/AP, E-CNC2/AP, E-CNC3/AP, and E-CNC4/AP. These samples were subjected to ultrasound treatments (frequency 20 kHz; amplitude 60%; power 450 W) applied for 300 s (with pulse mode durations of 2 s on and 4 s off).

We investigated the influence of solely APP (with no added CNC) on the physical stability of HIPEs. Our experimental results demonstrated that the absence of CNC led to an unstable emulsion system, characterized by a distinct phase separation. Therefore, we have ceased production of HIPEs without CNC, as their physical instability was not conducive to reliable performance.

### 2.3 Characterization of Pickering-HIPEs-Based Ink

#### 2.3.1 Emulsion Stability by Vertical Laser Profiling

The Turbiscan stability index (TSI) is a crucial parameter that determines the creaming stability of emulsions through multiple light scattering (S-MLS) experiments. By considering various storage processes of the emulsion, including particle coalescence and settling processes, we find TSI provides accurate results. We used a Turbiscan Lab Expert stability analyzer (Formulaction, Toulouse, France). The creaming stability of the emulsion was measured by vertical laser profiling for 180 min under ambient conditions. The creaming stability of the emulsion was evaluated through the application of pulsed near-infrared light (880 nm) and multiple light backscattering. The emulsions were placed in a test bottle with a height of 48 mm and monitored every 4 h for a total of seven intervals. The backscattering and transmission light (*T*) were recorded based on the Lambert–Beer theory, and the following formula was utilized^34^
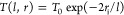
1The parameter *r*_i_ represents the inner diameter of the sample cell, while *T*_0_ denotes the transmittance of the continuous phase, namely, water. From a physical perspective, *l*(*d*, φ) was determined using the formula provided below^16^
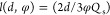
2This approach relied on the photon transport mean free path (*l*), mean droplet diameter (*d*), volume fraction of droplets (φ), and optical parameter (*Q*_s_) derived from Mie theory. The transmittance detector received light that had passed through the dispersion at a 180° angle from the source, while the backscattering (BS) detector received light scattered backward by the emulsion at a 45° angle
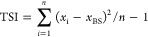
3In the given formula, χ_i_ represents the average backscattering for each minute during the experiment, χ_BS_ refers to the average χ_i_, and *n* denotes the number of scans.^16^

#### 2.3.2 Particle Size Measurement

The mean particle size and size distribution of HIPEs containing relatively small particles (*d* ≤ 400 nm) were measured by the dynamic light scattering technique using a Malvern Zetasizer (Nano-ZS; Malvern Instruments, Worcestershire, U.K.). To avoid multiple scattering effects, the emulsions were diluted with phosphate buffer solutions at a pH and/or salt concentration the same as the emulsion samples, before measurements.

The size and distribution of droplets greater than 6 μm in emulsions were analyzed using static light scattering with the Mastersizer 3000 from Malvern Instruments, based in Malvern, U.K. To ensure accurate results, the emulsions were again first diluted with buffer to avoid multiple scattering effects. The refractive index (RI) of the organic and aqueous phases was assumed to be 1.47 and 1.33, respectively. The mean droplet size was determined as the Sauter mean diameter (*d*_3,2_ = ∑*n*_i_*d*_i_^3^/∑*n*_i_*d*_i_^2^) using the full-size distribution range. To ensure precision, the measurements were conducted in quintuplicate with freshly prepared samples. The histograms in the droplet size figure were adjusted vertically to facilitate comparison using sizing data from the same initial droplet volume. Unless otherwise stated, the droplet sizes mentioned in the figures represent the Sauter mean diameter (*d*_3,2_).

#### 2.3.3 Emulsion Microstructure

The microstructures of the HIPEs were studied using confocal laser scanning microscopy (CLSM) utilizing a 60, 40, or 20× objective lens. To observe the layers, the collected creamed or top layers were stained with Nile red. Specifically, 100 μL of the collected sample underwent staining with the addition of 10 μL of Nile red solution (1 mg mL^–1^ ethanol), followed by gentle agitation. A 7 μL sample of the stained samples was then deposited onto a microscope slide and covered with a glass coverslip. The coverslip was rapidly secured with wax (Percheron Plastic, Inc., Canada) to prevent evaporation. Nile red’s excitation and emission spectra were measured at 488 and 539 nm, respectively.

A fluorescence microscope, specifically the Olympus BX53 with a 60× objective lens, was utilized to simultaneously identify the particles as well as the oil-dispersed phase. Before observation, Nile red was used to dye the oil phase, and Calcofluor white was used to stain the APP. The excitation and emission spectra for Calcofluor white in this study were 365 and 435 nm, respectively. Red and blue fluorescence signals were processed by using ImageJ code to generate merged images. In some cases, multiple droplets present in the sample created image overlapping, which could be mistakenly taken as a result of flocculation.

To examine the arrangement of oil droplets, we utilized in situ cryogenic scanning electron microscopy (SU8000 SEM device, Hitachi Co., Ltd., Tokyo, Japan). Our procedure involved freezing a small amount of the relevant emulsion in the specimen holder and submerging it in liquid nitrogen for around 20 s. Subsequently, we placed the sample in the Quorum cryogenic system’s preparation chamber attached to the scanning electron microscopy (SEM), which operated at a temperature of −140 °C. We assessed the emulsion’s structure by fracturing the sample with a blade (without coating) and using cryo-SEM at increasing temperatures in two stages: from −140 to −80 °C (5 min) and from −80 to −70 °C (20 min). We applied a platinum coating to the samples to aid in the observation.

#### 2.3.4 Rheological Experiment

An AR 2000ex rheometer by TA Instruments in New Castle, DE, was used to study the rheological properties of ink samples. The striated plate–plate geometry had a diameter of 40 mm and a gap of 1 mm equipped with a Peltier temperature controller. To determine steady rheological properties, the shear stress was measured at increasing shear rates from 0.1 to 500 s^–1^. The oscillatory strain sweep identified the linear viscoelastic region (*LVR*) using a range of 0.1–100% at 1 Hz. Additionally, the frequency sweep test (*ω*) was conducted in *LVR* (*γ* = 1%) at a range of 0.1–400 Hz. All measurements were performed at 25 °C, and rheological parameters like the elastic modulus (*G*′) and loss modulus (*G*″) were evaluated using the manufacturer-supplied computer software TRIOS by TA Instruments in West Sussex, U.K.

Thixotropic data on the emulsions was gathered by conducting a five-interval test using 5-ITT. The main objective of this test was to assess the Pickering-HIPEs’ ability to recover quickly when subjected to high deformations. A material that exhibits an ideal thixotropic structure should demonstrate a peak viscosity recovery of at least 70% of its value measured after the initial interval after 100 s. The samples were analyzed by 5-ITT, which detected their viscosity profiles under alternating high and low shear rates (80 or 0.1 s^–1^, respectively) for 100 s each.

#### 2.3.5 Creep and Creep-Recovery Test

Creep and creep-recovery measurements were performed to evaluate the compliance level during the creep and recovery stages via an AR 2000ex rheometer (TA Instruments, New Castle, DE). First, a stress sweep (1 Hz, 0.1–10 Pa) was accomplished (data not shown) to evaluate the oscillatory yield stress (*G*′(τ) = *G*″(τ)), and then the obtained values were considered as being about 50% of the yield stress. The HIPE-based inks were moved to a parallel-plate geometry with a diameter of 40 mm and a gap size of 1 mm, maintained at 25 °C. The creep measurement included the prompt application of a constant shear stress within the *LVR* area, lasting from 0 to 500 s, while evaluating the sample deformation during these time intervals. Regarding the recovery phase, the applied stress was rapidly removed (*τ*_applied_ = 0.0 Pa) and the recovery values were recorded for an additional 500 s at the same temperature as that in the creep phase.

#### 2.3.6 Nonlinear Rheological Response

A Fourier transform (FT) rheology method was applied to quantify viscoelastic nonlinearity, which is used in many types of complex fluids including polymer solutions and polymer nanocomposites. In this test, the stress signals were analyzed using FT rheology, which showed the total nonlinear viscoelastic stress. It could be separated into linear viscoelastic stress and odd higher harmonic contribution. The relative third harmonic intensity (*I*_3/1_) was found to be the most intense among the higher harmonics. At a small strain amplitude (*γ*_0_), *I*_3/1_ increased as a quadratic function of strain amplitude *γ*_0_. Using the equations suggested by Hyun et al. (2011), a nonlinear mechanical coefficient  and the intrinsic nonlinearity  (limiting value of *Q* at small shear strain) were calculated based on this scaling relation.

### 2.4 3D Printing of Pickering-HIPE-Based Inks

#### 2.4.1 3D-Printing Process

To produce 3D-printed Pickering-HIPEs, we began by preparing Pickering-HIPE-based inks. These inks were then used for printing via an extrusion-based 3D printer (nScrypt-3D-450, nScrypt, Orlando, FL) that was connected to a syringe pump (PHD Ultra; Harvard Apparatus Holliston, MA). To achieve a variety of unique 3D shapes, such as a one-dimensional (1D) zigzag pattern and two-dimensional (2D) square lattice, we utilized computer-aided design software (AutoCAD; Autodesk Inc., San Rafael, CA) to design the shapes and convert them to an STL file. The G-code files were used to control the XYZ direction of the printer and were created by using open-source CAM software called Slic3r, which was generated from the STL file. The printable Pickering-HIPE-based inks were poured into a 10 mL stainless steel cartridge and stirred with a Vortex mixer from Fisher Scientific in Ontario, Canada, for 15 min to remove air bubbles. The Pickering-HIPEs were then printed through a 1 mm needle with an extrusion flow speed of 25 mL min^–1^ at an ambient temperature on a special plastic surface. After printing, the resulting 3D-printed constructs were washed alternatively with absolute ethanol and deionized water to remove the internal phase. Finally, they were freeze-dried using Martin Christ’s α 2–4 LD Plus in Osterode am Harz, Germany, to yield Pickering-HIPEs.

#### 2.4.2 Microstructure Evaluations of 3D Structures

To produce highly detailed images with a deep field of view, the morphological structure of the 3D-printed objects was carefully examined using a field-emission scanning electron microscope (FE-SEM, S-4700, Hitachi, Japan). Before analysis, each 3D construct was precisely trimmed to dimensions of (15 × 15 × 15) mm^3^. To obtain the microstructures of the sectioned 3D-printed samples, a series of steps were followed. Initially, the samples were carefully mounted on a Peltier-cooled stage that maintained a temperature of −10 °C to prevent any thermal damage. Next, nitrous oxide was used as an imaging gas with a pressure of 50.7 Pa. Finally, each 3D construct’s microstructures were captured through a solid-state backscatter detector using an accelerating voltage of 20 kV.

#### 2.4.3 Mechanical Strength of 3D-Printed Objects

A mechanical assessment focusing on the tensile strength was conducted on dumbbell-shaped 3D structures featuring a 10 mm gauge length, 2 mm width, and 2 mm thickness. This evaluation was carried out at a cross-head speed of 100 mm/min using an Instron 3366 electronic universal testing machine (Instron Corporation, MA). The elastic modulus (*E*) of the 3D-printed samples was determined by calculating the average slope over the strain range of 10–30%, derived from the stress–strain curve. To examine the fracture energy (*Γ*) and the underlying toughening mechanism in the 3D-printed objects, additional evaluation procedures were employed.

To investigate the fracture process and toughening mechanism in the 3D-printed structures, two experimental procedures were implemented. First, each specimen underwent loading–unloading cycles under a tensile strain below their respective yielding strains. Subsequently, the specimens were subjected to successive and progressive stretches, where they were stretched to various strains during the first loading, relaxed to zero force, and then reloaded for the second loading. The ratios *E*_second_/*E*_first_ and *Γ*_second_/*Γ*_first_ were calculated to assess the impact of these stretches on the fracture process and the toughening mechanism in the 3D structures.

To examine the recovery behavior of the notched samples, a loading–unloading cycle at a fixed strain (*ε* = 400%) was performed initially. Following this, the deformed and relaxed notched samples were enclosed in a polyethylene bag and preserved in a water bath maintained at 37 °C. At different time intervals, the specimens were retrieved from the bath, cooled to room temperature, and subjected to tensile tests once more. This series of experiments aimed to understand the recovery characteristics of the 3D-printed objects under the specified conditions.

#### 2.4.4 *In Vitro* Curcumin Release Test and Scaffold Disintegration

Freeze-dried 3D-printed capsule with dimensions 40 mm  ×  10 mm × 10 mm (length  ×  width  ×  height) or self-supporting circular mesh with dimensions 50  mm × 30  mm (diameter ×  height), containing curcumin (a model drug) (*n* = 3), were weighted and then soaked in 40 mL of phosphate buffer saline (PBS) solution (pH 7.4) at 37 °C with constant stirring for 24 h. The released curcumin was then measured. In brief, at predetermined times, PBS was completely withdrawn and centrifuged for 30 min at 14,000 rpm. PBS was dismissed, whereas the pellet inks and curcumin were resuspended in 15 mL of ethanol. The amount of released curcumin was determined at room temperature by an ultraviolet–visible–near-infrared spectrophotometer (UV–vis–NIR, Shimadzu 3600, Japan), comparing the absorbance at *λ* = 426 nm (maximum absorbance of curcumin) with a standard curve. The standard curve was constructed by measuring the absorbance at known concentrations of curcumin (from 0 to 0.025 mg mL^–1^) prepared in ethanol. The results were calculated as follows
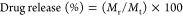
4where *M*_r_ is the amount of curcumin released at different times and *M*_t_ is the total amount of curcumin loaded in the printed scaffold.

Considering that curcumin is completely soluble in ethanol, whereas ink tends to precipitate, the curcumin-loaded scaffold of each sample was stored to quantify the amount of disintegrated ink from the printed part. For that purpose, the solution was centrifuged for 30 min at 14,000 rpm to collect the precipitates separately. These were dried and weighed and then compared to the initial weight of the scaffold, following eq 4 as previously described.

### 2.5 Statistical Analysis

All instrumental experiments were carried out in triplicate, and the mean and standard deviation of the data were reported. Analysis of variance (ANOVA) was utilized to determine the main effects of the examined independent factors and their interactions with the instrumental data. Duncan’s multiple range test was applied to separate means of data when significant differences (*p* < 0.05) were observed.

## 3 Results and Discussion

### 3.1 Morphological and Structural Characterization Studies of CNC and APP

**Figure 1:**
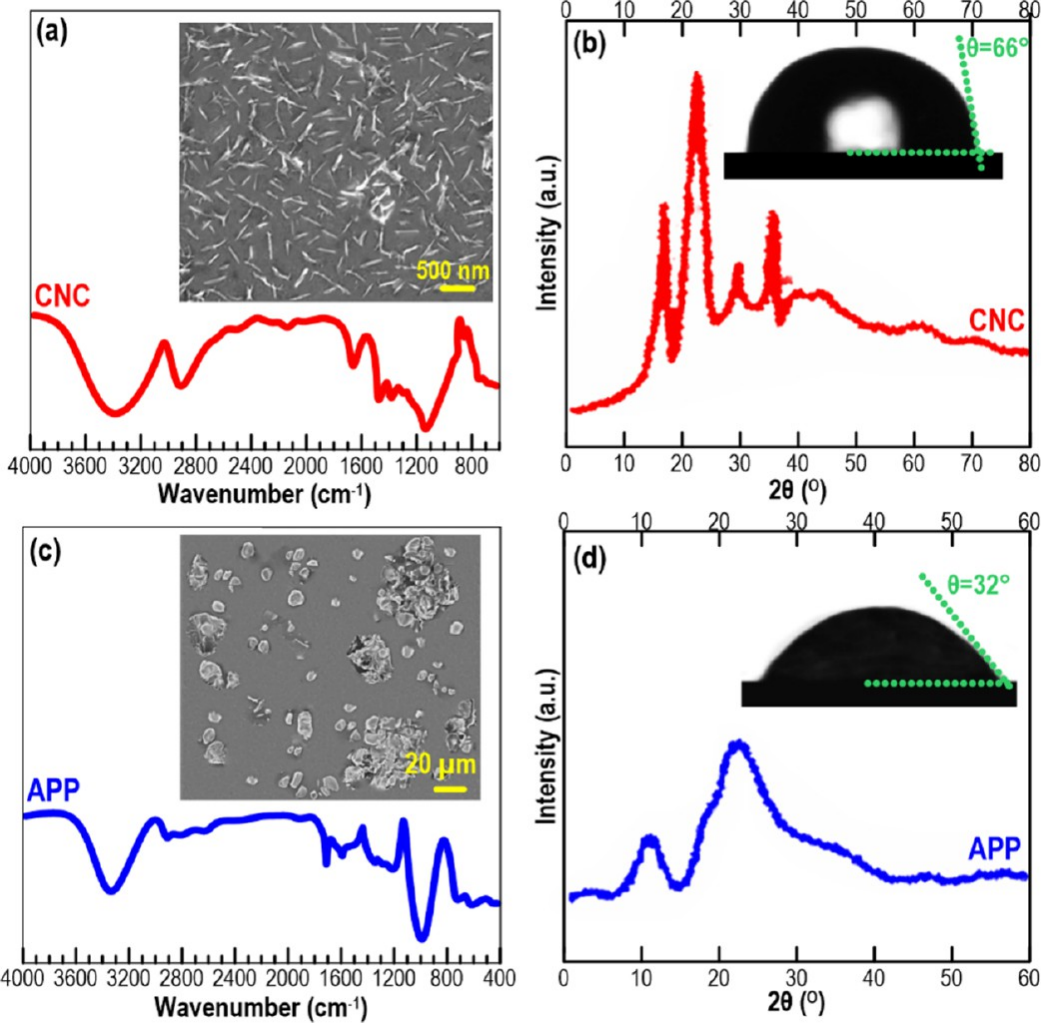
Structural and morphological characterizations of CNC^43^ or APP. (a, c) FTIR and related SEM images and (b, d) X-ray diffraction (XRD) and water contact angle measurements.

As CNC and APP played an important role in this work, the chemical structures and morphologies of pure CNC and APP were characterized by Fourier transform infrared (FTIR) spectroscopy and SEM evaluation (Figure 1). The FTIR of CNC was characterized by cellulose type-*I*, centered from 2500 to 3750 and 700 to 1800 cm^–1^ (Figure 1a). In this case, the stretching of O–H emerged at 3430 cm^–1^ followed by some typical peaks around 2960 cm^–1^ (related to C–H stretching), 1655 cm^–1^ (associated with asymmetric stretching of a carboxyl group), 1455 cm^–1^ (assigned to methylene symmetrical bending), 1108 cm^–1^ (allocated to cellulose C–O–C bridge), and a peak around 880 cm^–1^ that corresponds to β-linked glucose moieties. The microstructure of CNC was needle-shaped, ranging from 100 to 700 nm in length and approximately 10–30 nm in diameter (Figure 1a). The diffractogram of pristine CNC presents a crystalline structure with a relative crystallinity of 71% (Figure 1b), with the dominance of cellulose type-*I* having characteristic peaks around 2θ = 16.5, 22.5, and 33°. These can be assigned to the planes (110), (200), and (004), respectively. A model thin film CNC was made by the spin-coating approach (KW-4A spin-coater, CHEMAT Technology Northridge, CA), and then a sessile drop water contact angle measurement was performed.^35^ The water contact angle of the CNC-based film was found to be θ = 66° (Figure 1b), which is likely due to its high level of crystallinity degree and the presence of nanoscale topography.

The FTIR of APP presents a wide peak from around 3600 to 3100 cm^–1^, which is associated with the O–H and N–H bond stretching (Figure 1c), existing in lignocellulose components of APP.^36^ The peak present around 2850 cm^–1^ relates to both symmetric and asymmetric stretching of C–H bonds. The peak around 1750 cm^–1^ is associated with the stretching absorption of carbonyl, which can also be induced by the C=O in lignin and hemicellulose.^36^ The SEM image of the APP seemed to be fairly round particles with no sharp edges. There is a mix of small (2 μm) and big particles (40 μm), while some clusters are also evident (Figure 1c). The diffractogram of APP also shows two characteristic peaks around 2θ = 10.5° and 2θ = 22.5° with a relative crystallinity of 56% (Figure 1d). The water contact angle experiment revealed that the APP model film prepared by the spin-coating method offered a high hydrophilic character, presenting a low water contact angle of about θ = 32° (Figure 1d). In this sample, the water droplet was completely absorbed into the APP-based film after 60 s. The APP backbone is thus quite wettable by the aqueous phase because of the presence of a large number of hydrophilic groups.^31^

### 3.2 Characterization of Pickering-HIPE-Based Inks

#### 3.2.1 Interfacial Adsorption and Interfacial Rheology Behaviors

**Figure 2:**
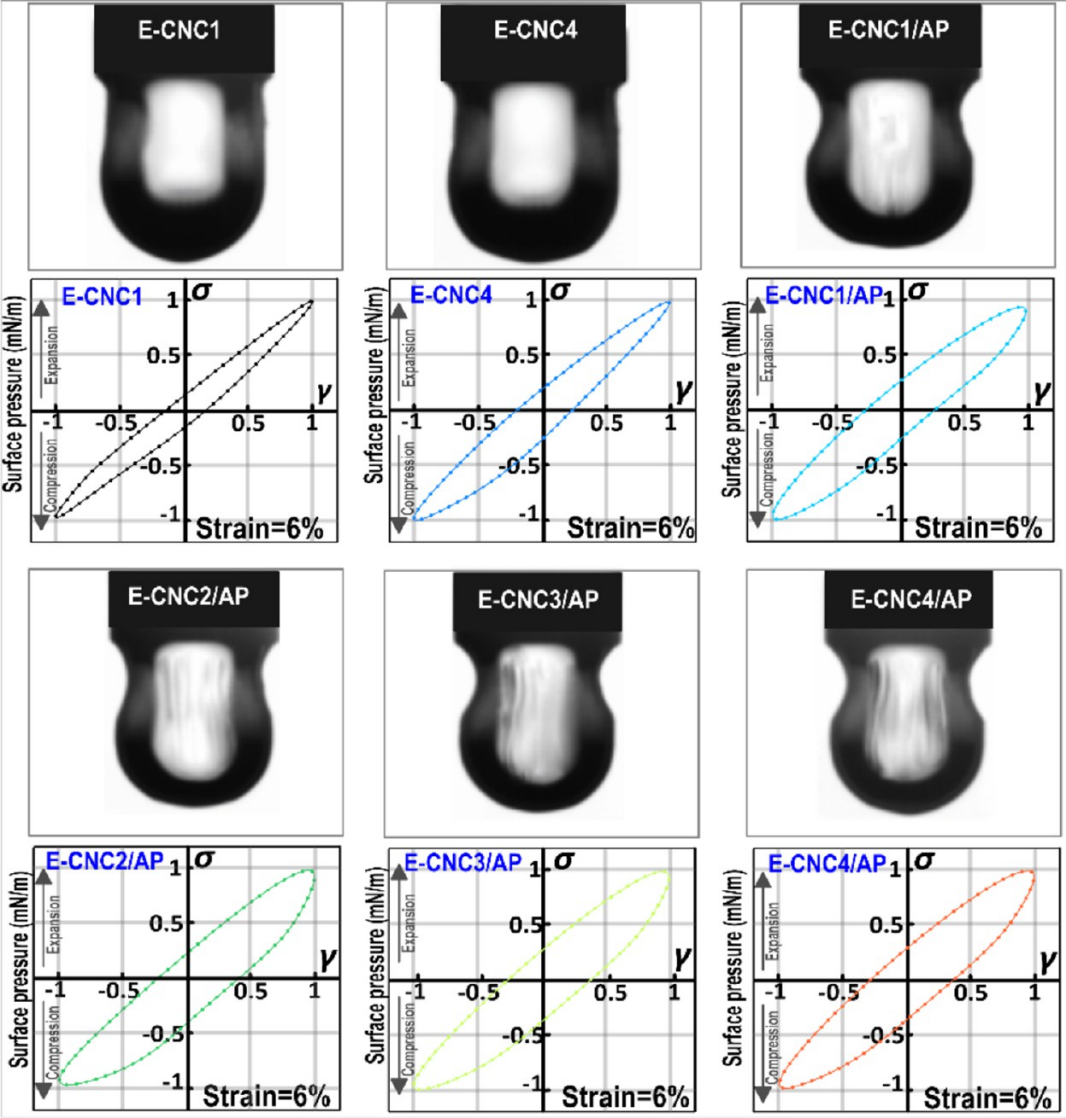
Dynamic interfacial tension of the isopropyl myristate–water interface with the related elastic and viscous modulus of interfaces of different HIPE-based inks.

To further elucidate the adsorption mechanism of CNC particles to the surface of droplets, the “pendent drop” method was utilized to measure the dynamic interfacial tension and observe the drop appearance during deformation. Figure 2 shows the existence of wrinkles on the droplet surface containing CNC and APP during volume shrinkage. The wrinkling of the skin-like interfacial structure became more evident as the CNC content was increased. The better emulsification of higher levels of CNC in the presence of APP could be associated with its higher interfacial coverage and also increase the rigidity of the interface. Furthermore, Figure 2 also depicts relevant Lissajous curves of the above interfaces, showing a well-defined symmetry, reflecting that the amplitude value of 6% was inside the linear viscoelastic region. The surface pressure was greater at the identical deformation with increasing CNC content in the presence of APP.

**Figure 3:**
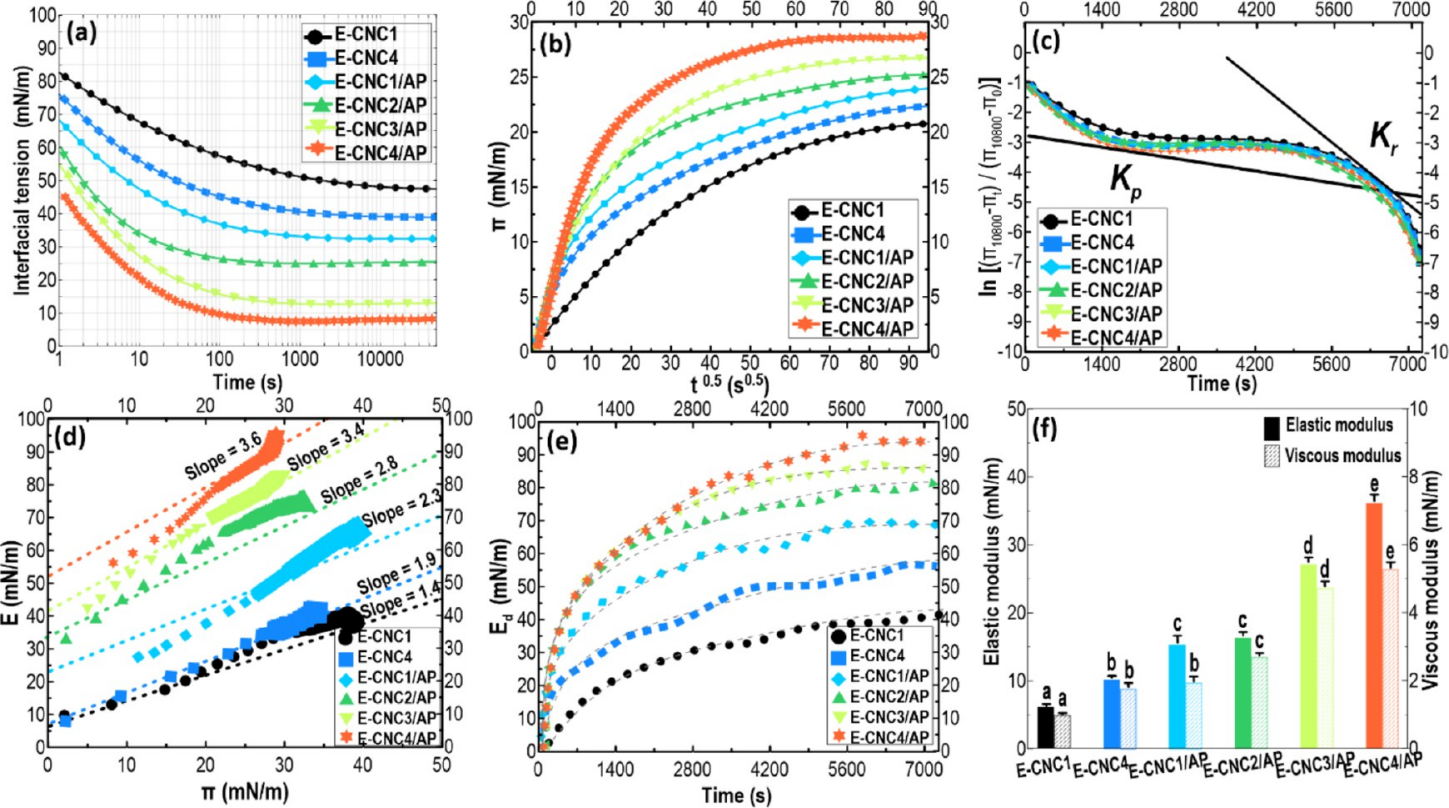
(a) Interfacial tension, (b) time dependence of surface pressure (π), (c) typical profile of the molecular penetration and configurational rearrangement steps at the O/W, (d) surface dilatational modulus (*E*) as a function of surface pressure (*π*), (e) time-dependent dilatational elasticity (*E*_d_) as a function of time, and (f) elastic and viscous modulus of interfaces. Error bars in (f) represent standard deviation. Moreover, different letters in each column indicate significant difference (*p* < 0.05).

Figure 3a also shows that the interfacial tension of particles rapidly reduced in the early phase, especially for CNC-contained HIPEs with APP, which is likely assigned to rapid adsorption of CNC at the O/W interface when APP exists. On the whole, the functionalities of solid particles at an O/W interface contain three processes: diffusion, penetration, and reorganization. In this case, the dynamic interfacial tension reduced when higher CNC levels were used, albeit in the presence of APP (Figure 3a). Consistent with Ward and Tordai’s calculation, the adsorption of particulate-type emulsifiers at the O/W interface depends on diffusion-controlled adsorption.^40^ It has been stated that APP possesses emulsification properties of both particles and surfactants.^38,41^ Thus, it is possible that the adsorption of CNC-containing HIPEs comprising APP at the interface could not only decrease the interfacial free energy but also develop a steric barrier to colloidally stabilized HIPE systems.

The interfacial pressure and viscoelasticity of CNC/APP at the O/W interface were evidenced to efficiently affect the emulsifying ability of the Pickering-HIPEs. Dilatational rheology was employed to investigate the interfacial properties of CNC-containing HIPEs with and without added APP to better monitor the relationship between the interfacial behavior and emulsification of CNC/APP. The interfacial surface pressure (*π*) as a function of the square root of time (*t*^0.5^) is shown in Figure 3b. Regardless of the sample type, a quick increase in *π* was noticed in the initial phase of plots, which specified that the adsorption process was diffusion-controlled during this stage. A linear fitting was then used to determine the rate of diffusion (*K*_diff_) (Table 1). With an increase in the CNC content in the HIPEs containing APP, there is an increase in the *K*_diff_ value. For example, the *K*_diff_ value of E-CNC1/AP increased from 0.229 to 0.636 mN/m/s^0.5^ regarding E-CNC4/AP. This reveals that in the presence of APP, the diffusion process was controlled by the concentration effects at the higher CNC content. This can then propose that the addition of APP in the CNC-contained Pickering-HIPEs endowed a favorable impact on the diffusion–adsorption process of CNC. Thus, the emergence of aggregated particles and/or aggregated networks slows down the adsorption rate of the solid particles at the O/W interface.

**Table 1:** Characteristic Dynamic Parameters of Adsorption on the O/W Interface at the End of Adsorption (*π*_10800_) for Different Samples^a^

sample	*K*_diff_ (mN/m/s^0.5^)	*K*_p_ × 10^–5^ (linear regression)	*K*_r_ × 10^–5^ (linear regression)
E-CNC1	0.067 ± 0.004^a^	–2.551 ± 0.067^a^	–14.43 ± 0.021^a^
E-CNC4	0.073 ± 0.003^b^	–2.112 ± 0.031^b^	–9.89 ± 0.014^c^
E-CNC1/AP	0.229 ± 0.006^c^	–2.103 ± 0.049^b^	–10.78 ± 0.029^b^
E-CNC2/AP	0.380 ± 0.011^d^	–1.767 ± 0.042^c^	–8.36 ± 0.058^d^
E-CNC3/AP	0.567 ± 0.012^e^	–1.455 ± 0.063^d^	–7.34 ± 0.069^e^
E-CNC4/AP	0.636 ± 0.015^f^	–1.149 ± 0.041^e^	–7.07 ± 0.089^e^

a All data were expressed as mean ± standard deviation, and different letters in each column indicate significant differences between samples (*p* < 0.05). *K*_diff_, *K*_p_, and *K*_r_ refer to the diffusion rate, penetration rate, and reorganization rate, respectively.

Furthermore, the rearrangement and penetration rates of an adsorbed layer at the O/W interface were assessed by a first-order equation.^40^ In Figure 3c, the slope curve of ln [(*π*_10800_ – *π*_t_)/(*π*_10800_ – *π*_0_)] is plotted against time. Two linear regions are noticeable, showing the penetration (*K*_p_) and reorganization rates (*K*_r_) of our particulate emulsifiers into the interfacial film. According to this plot, the solid particles experienced a reorganization and penetration process at the O/W interface. Table 1 summarizes the *K*_p_ and *K*_r_ in which all samples possessed negative *K*_r_ and *K*_p_ values, and the absolute value of *K*_r_ was higher than that of *K*_p_. The absolute values of *K*_p_ slightly decreased with an increasing CNC content, whereas the absolute values of *K*_r_ notably increased. The obtained results already verified that the rearrangement rate of CNC/APP was a little affected, yet the penetration rate was meaningfully enhanced. The result was consistent with the previous observations of the relevant Pickering emulsions at the O/W interface.^14,42^

To characterize the mechanical features of the interfacial layer, the dynamic viscoelastic property of the adsorbed layer at O/W interfaces can be related to surface dilatational modulus (*E*). This is commonly measured by a change of dilatational stress (interfacial tension, γ) resulting from a slight alteration in surface area (dilatational strain).^43^Figure 3d illustrates the plots of *E*–*π* for the interfacial layer. As can be observed, with the increase of *π*, the *E* increased at any test concentration, showing the continuous adsorption of solid particles at the interface. Furthermore, the slope of *E*–*π* curves at any specific concentration was greater than 1, highlighting the nonideal properties of the molecular interactions between particles adsorbed at the interface.^14^ Alternatively, Figure 3e presents the adsorption time dependence of the dynamic dilatational elastic modulus (*E*_d_) of interfacial layers. Regardless of the sample type, the *E*_d_ values increase as a function of time and finally reach plateau. This reflects a fast adsorption process followed by saturation. In the current work, the increase of *E*_d_ was affected by CNC contents, yet the sample formulated by APP suggests that the adsorption and diffusion processes could be effectively improved by the presence of APP. Consistent with the data of interfacial dilatational moduli (Figure 3e), when APP was added to the system, the higher contents of CNC offered an interfacial film with higher elastic and viscous properties (Figure 3f). Bearing in mind the above, the higher viscoelastic features of droplet interfaces could be likely associated with greater interfacial coverage as affected by the adsorption of CNC and more distinctively APP, thus providing a more robust interfacial film.^44^

#### 3.2.2 Flocculation of Pickering-HIPEs

**Figure 4:**
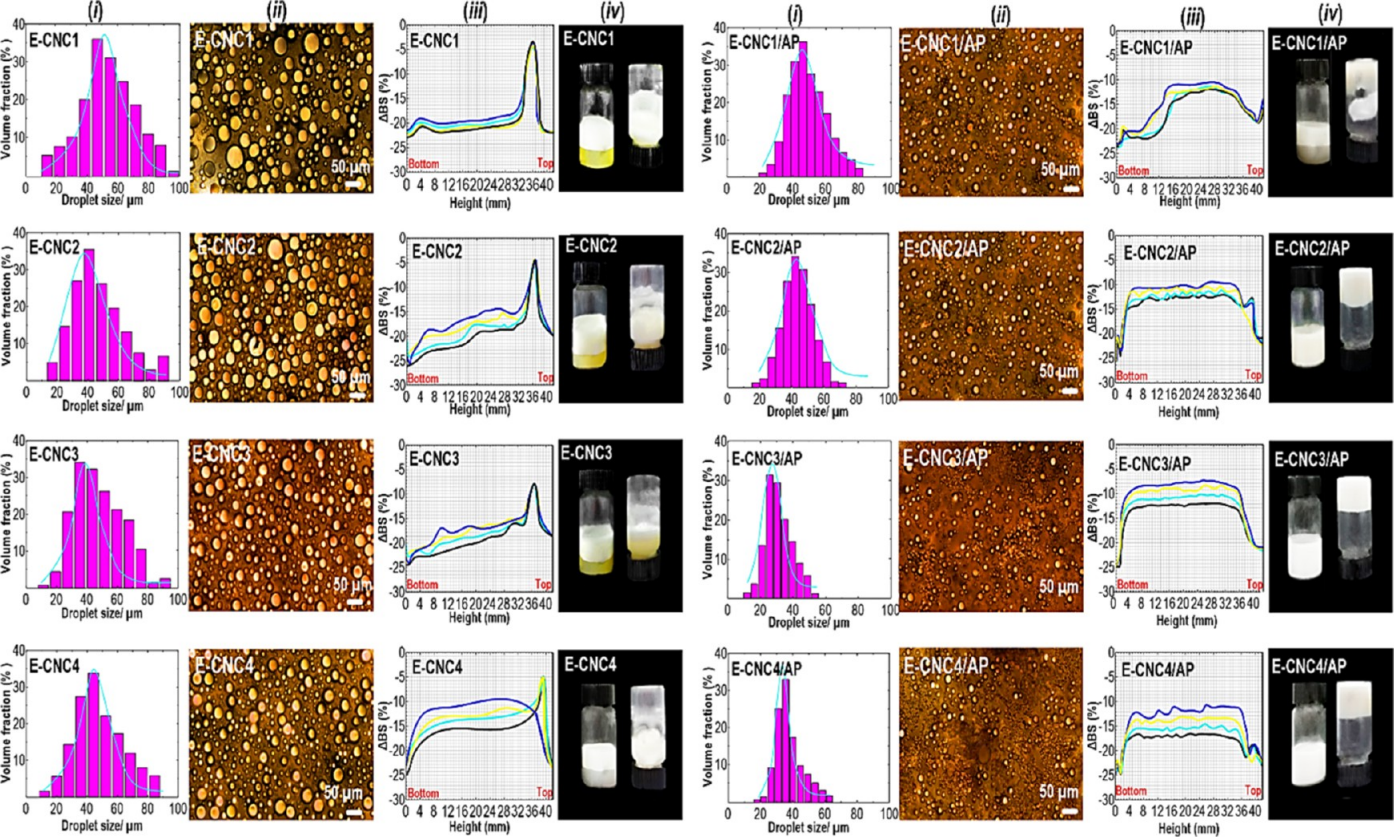
(*i*) Droplet size distribution, (*ii*) microscopic images, (*iii*) backscattering intensity profiles provided by Turbiscan (in each curve, the blue, green, cyan, and black lines represented the different storage times 24, 48, 72, and 96 h), and (*iv*) visual observation of CNC-contained printing of HIPE-based inks without (left) and with (right) added APP.

The droplet size distribution, light microscopic images, multiple light scattering measurements, and visual observation of printing HIPE-based inks formulated with CNC and CNC/APP are illustrated in Figure 4. The droplet size distribution for different emulsions as a cumulative function of the average of three consecutive measurements was strongly affected by the presence of solid particles. In this case, Figure 4*i* (left) displays the droplet size distribution of HIPEs prepared with different CNC contents with no added APP, whereas Figure 4*i* (right) illustrates the droplet size distributions for samples also containing 1.5 w/w APP (Section S.1). Taking a comprehensive look at the distribution curves of the emulsions, it was detected that with increasing CNC content, the droplet size was slightly reduced (Figure 4*i*, left). More specifically, the distribution curve became somewhat narrower and more uniform for the HIPEs with higher levels of CNC. The observed outcome can be attributed to the increased surface coverage by CNC. This increased surface coverage effectively prevents droplet coalescence, thereby resulting in a smaller droplet size.^37^ Alternatively, a notable decrease in emulsion droplet size was observed after the incorporation of APP into the CNC-containing HIPEs, where the distribution curves are seen to shift to lower sizes. It is clear that the proportion of the smaller-sized population was increased in Pickering-HIPEs with the higher CNC concentrations that also included APP (Figure 4*i*, right). The large-sized population (>10 μm) was also absent in these latter emulsions, while it was very noticeable in Pickering-HIPEs with no added APP. Therefore, it seems that the incorporation of APP is responsible for the improved colloidal stability of emulsions against coalescence. Despite the scarcity of research on the stabilization of HIPEs using “raw” submicron-sized sustainable particles, there is an existing body of literature on the use of these compounds in traditional emulsions, where they have been shown to inhibit coalescence even at low surface coverage concentrations.^38^ It is hypothesized that the stabilization process of this particle is likely the result of a combination of mechanisms arising from the presence of both soluble compounds and solid particles.

An optical light microscope was used to observe and illustrate the microstructures of diverse HIPE-based inks, as displayed in Figure 4*ii*. For all CNC-containing Pickering emulsions, a prominent feature was the presence of spherical structures consisting of oil droplets with relatively large sizes (Figure 4*ii*, left). As expected, the spherical oil droplets are surrounded by smaller solid CNC particles. In the absence of APP, it is evident that lower CNC concentrations result in smaller droplet sizes. As the CNC content increases, the droplet size decreases, which may represent the fact that more surface area can be stabilized by a higher amount of CNC. The microscopic observations of the emulsion droplets align with the findings of their droplet size distribution reported above. The effect of APP addition on the droplet size of CNC-containing Pickering-HIPEs is also shown in Figure 4*ii* (right). After the incorporation of APP, typical flocculation and aggregation of emulsion droplets were identified. However, noteworthy that despite being aggregated, the actual droplets themselves were of a smaller size when APP was present. Concerning samples with higher CNC content *(i.e.*, E-CNC3/AP and E-CNC4/AP), a high level of flocculation and aggregation was observed. Nonetheless, the size of actual droplets became smaller on the whole, when compared to E-CNC1/AP and E-CNC2/AP. This droplet flocculation—or aggregation—is frequently encountered in emulsions stabilized by “raw” microsized sustainable powder byproducts.^38^ Consistent with a recognized principle, the high internal-phase volume develops the aggregated and densely structured particles among the closely packed droplets, which can effectively form a gel-like structure. This structured particle-based network, created at the O/W interface and extending to within gaps between the close-packed droplets, is anticipated with CNC and APP, so long as the content of dispersed phases in this system is adequately high to form such a network. In fact, all of the interfacial particulate stabilizers (including surfactants, polymeric emulsifiers, and particulate stabilizers) can stabilize a gel-like HIPE at a high enough concentration. However, only the Pickering-HIPEs still behave similarly to a gel at a very low (even the lowest) particulate emulsifier (solid particles) due to large-scale flocculation. In the current work, the obtained results verified that even if the APP level was as low as 1.5 w/w, the produced printing HIPE-based inks still showed a gel-like morphology, benefited by APP-induced depletion flocculation (Figure 4*ii*, right). Prior studies revealed that the gelling behavior of emulsions can benefit from their Pickering nature, combined with some form of flocculation, e.g., resulting from bridging, as induced by APP between adjacent interfaces.^32,38^ As a whole, the incorporation of APP into the CNC-included Pickering-HIPEs develops a “floc” system formed from droplets of a lower size. This signifies a synergistic effect of CNC and APP on the HIPEs when deployed for producing 3D-printing ink formulations.

Additional information about the colloidal stability mechanism of the printing HIPE-based inks was accomplished by static multiple light scattering (S-MLS) measurements through the evaluation of particle size changes and particle migration phenomena. Figure 4*iii* shows a characteristic creaming profile representing different detected stability states. The *X*-axis signifies a height or distance from the base of the sample, while the *Y*-axis represents the backscatter intensity, which is a function of the size and number of scattering droplets or particles. Creaming can be detected by a decrease in backscattering intensity at the left side of the plot (the sample base) with a simultaneous intensity increase at the right side (top of the sample). Moreover, a decrease in the backscattering intensity at the center of the sample denotes the presence of flocculation. This is because the droplets move closer together and hence are more concentrated here. As a result, the light scattering is reduced because of fewer scattering centers in this region. The backscattering profile of Pickering-HIPEs relates to the optical light microscopy results, with the various levels of CNC exhibiting different general trends. The backscattering profiles were separated into different categories depending on the CNC contents. Initially, there was no flocculation when HIPEs contained a lower amount of CNC (i.e., E-CNC1 and E-CNC2), albeit with a higher degree of creaming detected in such samples (Figure 4*iii*). This denotes the fact that the backscattering intensity in the centers of these emulsions remained rather constant, while at the same time there was an increase in the scattering at the top part of the sample (the right-hand side of the graph). This behavior can be attributed to an upward movement of the individual oil droplets under the influence of gravity due to their lower density in comparison to the surrounding aqueous phase. On the other hand, as the CNC content increased (i.e., E-CNC3 and E-CNC4), the backscattering intensity in the central part increased noticeably, and there was a slight increase in intensity at the top of the sample compared to E-CNC-1 and E-CNC2. This effect can be attributed to the fact that the system is still highly unstable to creaming; i.e., the bigger droplets still do rise to the top. This means an increase in the volume fraction of droplets accumulating near the top because of their upward movement from the bottom of the tube. This is most likely due to the absence of a sufficient gel-like structure, and the overall low viscosity of the system arising in a well-dispersed system. As droplets are not part of a network and are free to move, their upward creaming is not being hindered.^39^

Alternatively, the addition of APP into the CNC-contained Pickering-HIPEs resulted in a flocculation process with no detected creaming, which was observed by a reduction of the backscattering intensity in the center of the graph, yet a slight alteration at the top or bottom of E-CNC1/AP and E-CNC2/AP (Figure 4*iii*). This effect may be associated with the fact that synergic effect of CNC (especially at the higher levels) and APP species on the network strength may efficiently increase the viscosity of the system to inhibit creaming of the flocculated droplets. Finally, the combined effect of added APP and CNC at the highest contents could inhibit the creaming phenomenon, i.e., the backscattering profile remained constant during storage. Figure 4*iv* (right) also showed that the CNC-contained Pickering-HIPEs with APP could hold their weight once the tubes were inverted at ambient conditions. This suggests that these samples were structured with a gel-like behavior, possessing a finite yield stress, and thus very stable against creaming. The physical stability of these Pickering-HIPEs was further confirmed by the backscattering data from the Turbiscan analysis (Figure 4*iii*). Indeed, the flocs’ movement is delayed in a structured emulsion system with increasing aqueous phase viscosity up to a point where the viscosity is sufficiently high that it limits the individual oil droplets’ movement. Thus, in the highly depletion-flocculated HIPE system, they could approach each other. In the Pickering-HIPEs with both CNC and APP, a high level of nonadsorbed particles can develop a high depletion force between droplets. This case is one of the strong benefits of Pickering-HIPEs that they can be flocculated but in which the droplets still do not coalesce.

#### 3.2.3 Morphological Evaluation of Printing Pickering-HIPE-Based Inks

**Figure 5:**
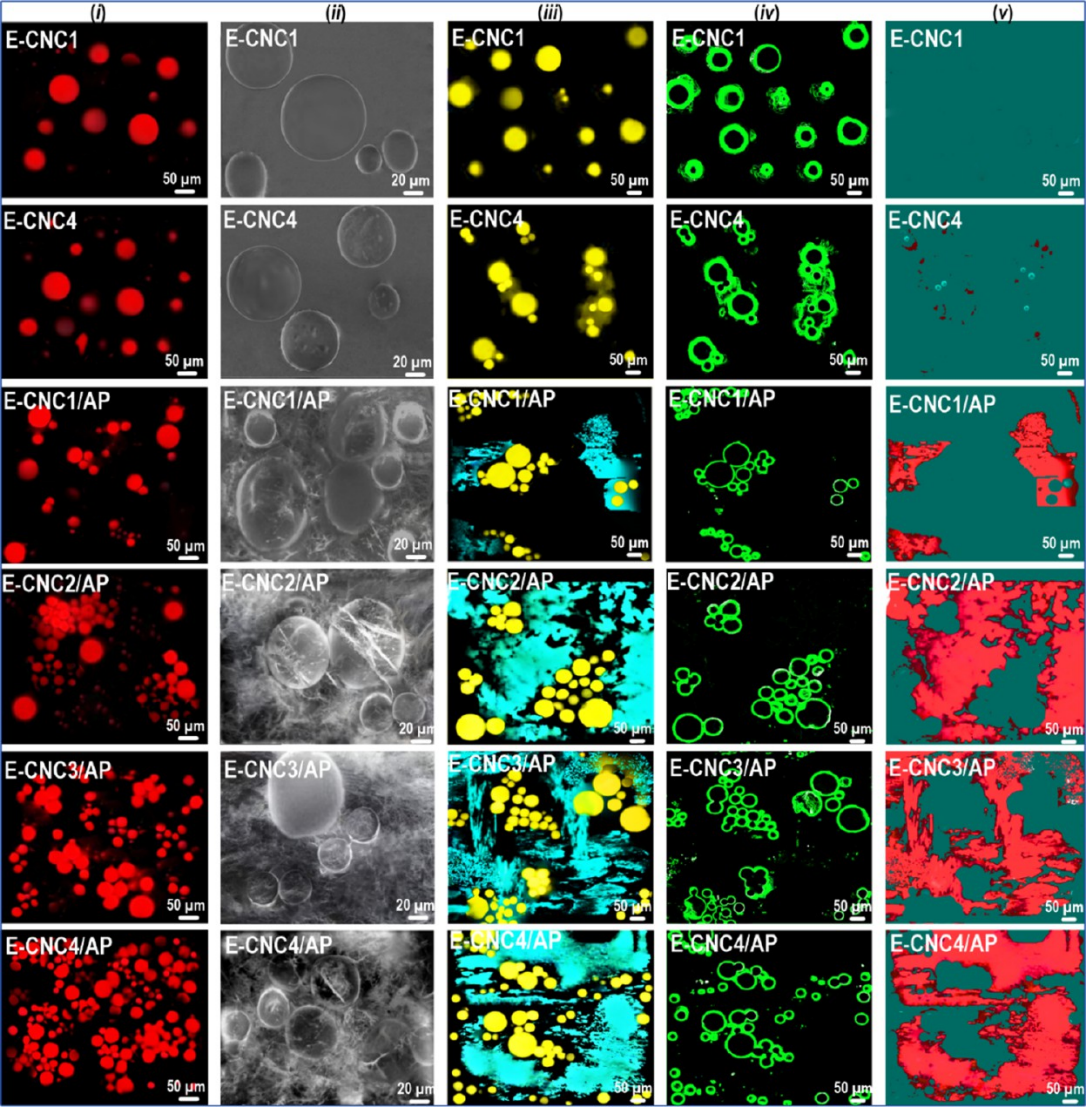
(Column *i*) CLSM (the oil phase was stained with Nile red before evaluation), (Column *ii*) cryo-SEM, (Column *iii*) results from the merged images fluorescent micrographs, (Column *iv*) fluorescent micrographs-stained oil phase, and (Column *v*) fluorescent micrographs-dyed APP of the top or creaming layer of different printing Pickering-HIPE-based inks.

Additional evidence supporting the inference that APP performed like a Pickering stabilizer is presented below. The microstructure of printing HIPE-based inks was imaged through a confocal microscope, which illustrated an obvious sign of droplet flocculation in the emulsion containing APP (Figure 5, Column *i*). However, E-CNC1 and E-CNC4 show somewhat comparable microstructures: a dispersed oil phase in a continuous water phase with no apparent signs of flocculation. In contrast, a flocculation phenomenon was detected in the Pickering-HIPEs with APP due to the presence of large and dense aggregates comprising oil droplets. By increasing the CNC content in these systems, the degree of flocculation was increased. Compared with E-CNC1/AP and E-CNC2/AP, the aggregated droplets of E-CNC3/AP and E-CNC4/AP also possessed a lower droplet size. In the flocculated emulsions, there were two very different droplet size types; the first below 5 μm and another about 20 μm, which agree well with the distribution size curve (Figure 5i, right). Further, E-CNC1/AP showed restricted droplet association because of possible bridging flocculation due to the inadequate amount of biosurfactant presented to cover a newly generated O/W interface during emulsification. As an alternative, other Pickering-HIPEs convert to a physically stable system related to a depletion effect. To sum it up, the confocal images verified that depletion interaction was developed by APP in Pickering-HIPEs with higher content of CNC (i.e., E-CNC3/AP and E-CNC4/AP), which was generic and happened in a wide range of droplet sizes, albeit offered different droplet dynamics. In particular, the CNC content and the presence of APP were key drivers defining the colloidal stability of printing Pickering-HIPE-based inks.

**Figure 6:**
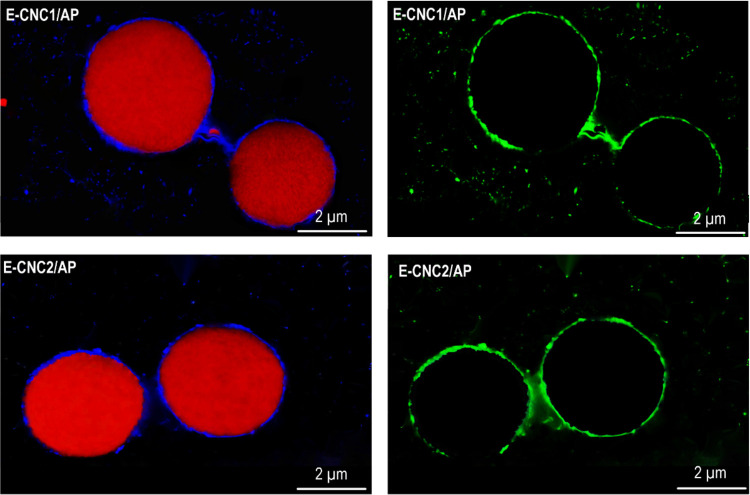
CLSM micrographs of E-CNC1/AP and E-CNC2/AP, showing the formation of possible bridging flocculation.

Given the microstructure monitoring of printing Pickering-HIPE-based inks, it is assumed that the dynamics of droplet coarsening, flocculation, and coalescence can be driven by the depletion phenomenon (or even bridging flocculation at a lower CNC concentration), occurring at a certain CNC content threshold. To verify the flocculation process, an experiment was designed to recognize the position of the particulate emulsifier in the Pickering-HIPEs (Figure 5, Columns *ii*–*v*). The interfacial framework and microstructure of Pickering-HIPEs were assessed with cryo-SEM images associated with the aqueous phase near the droplets (Figure 5, Column *ii*). For E-CNC1/AP, bridging flocculation was obviously detected with irregular compact APP being distributed surrounding the continuous phase (Figure 5, Column *iii*), while there was the occurrence of depletion flocculation, forming a network in which aggregated oil droplets are embedded. Based on fluorescent micrographs (Figure 5, Columns *iii*–*v*), it can be concluded that APP was present as nonadsorbed and adsorbed entities, developing the rigid interfacial layer covering the surface of droplets. This efficiently inhibited coalescence with time at rest. As a whole, bridging flocculation was observed in HIPEs with low levels of CNCs costabilized with AP (i.e., E-CNC1/AP and E-CNC2/AP) (Figure 6), while the depletion interaction was observed in HIPEs with higher CNC concentrations in the presence of AP (E-CNC3/AP and E-CNC4/AP).

#### 3.2.4 Flow Curves of Printing Pickering-HIPE-Based Inks

**Figure 7:**
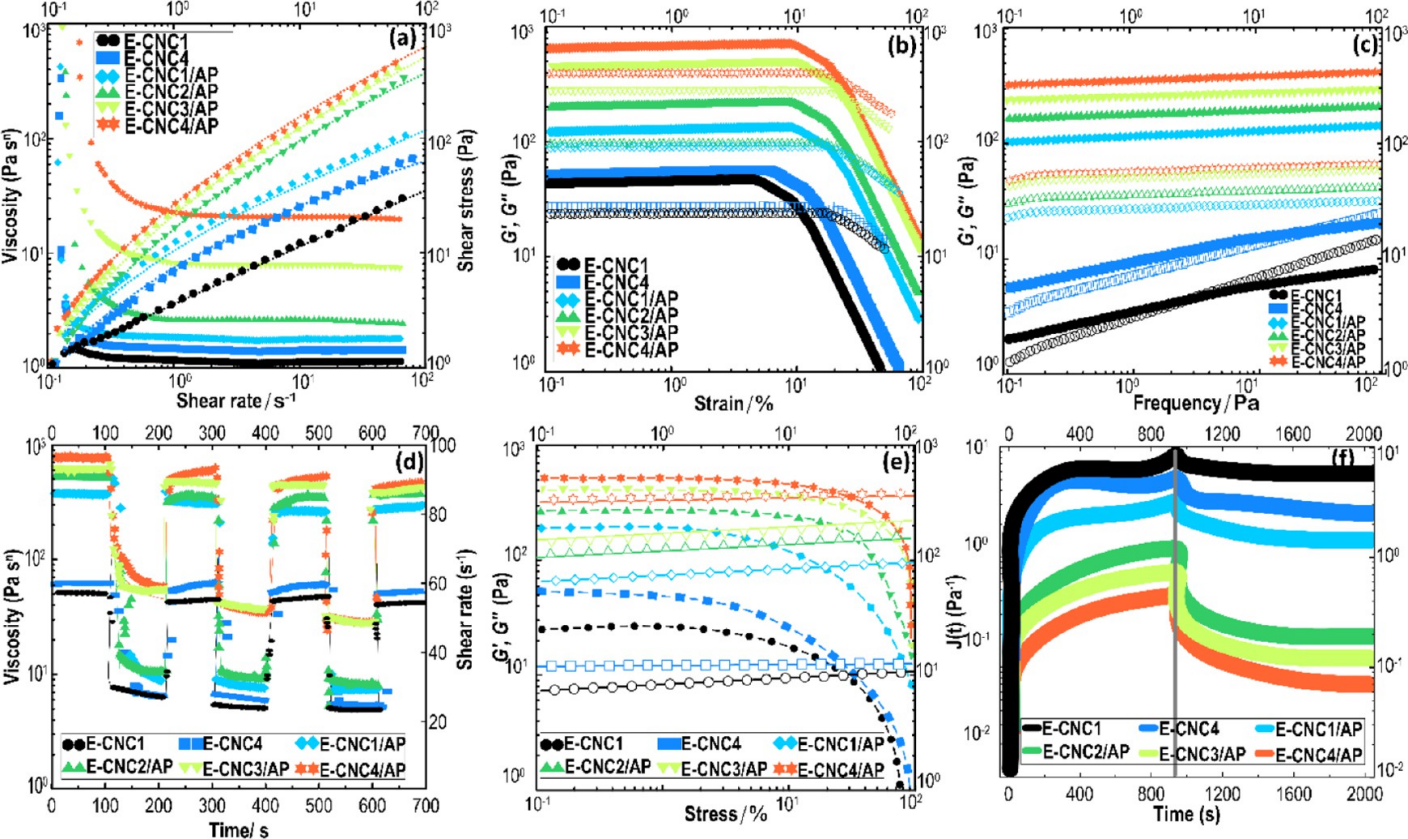
(a) Flow curve presents changes in the shear stress and viscosity with shear rate. (b) Strain sweep showing the linear viscoelastic region (*LVR*), and (c) frequency sweeps, where *G*′ is denoted by solid symbols and *G*″ is denoted by open symbols, (d) 5-ITT, (e) stress sweep with *G*′ and *G*″ considered as solid and open symbols, respectively, and (f) creep and creep-recovery curves of Pickering-HIPE variants.

To further clarify the effects of CNC and APP on the colloidal stability of printing Pickering-HIPE-based inks, the rheological properties of CNC-containing Pickering-HIPEs, both with and without added APP, were evaluated and compared. In this regard, the dependence of apparent viscosity and shear stress on the applied shear rate is shown in Figure 7a. The flow curve showed that the CNC-containing Pickering-HIPEs possessed shear-thinning behavior over the shear rate range from 0.1 to 100 s^–1^ with low-shear viscosity increasing with the CNC content. They also had a higher apparent viscosity at a low shear rate, which reduced significantly when the shear rate increased. As the shear rate increases, the droplet flocs can no longer support the applied stress. The gel-like structure is finally disrupted,^45^ and the oil droplets become more ordered along the flow field. The shear-induced smaller sized aggregates and this order together endow less resistance to flow; therefore, viscosity is reduced at higher shear rates (shear thinning). In the CNC-containing Pickering-HIPEs with no added APP, a slight increase in the apparent viscosity, detected with increasing CNC content (especially E-CNC4), could be due to a low level of flocculation in the dispersed oil phase. This was already noted in the CLSM images (Figure 5), where the CNC seemed to form some aggregates in the bulk oil phase at the interface. Thus, as expected for a weak flocculated system, a low degree of shear-thinning behavior was expected.^46^ A similar shear-thinning trend was found regarding CNC-contained Pickering-HIPEs with APP, yet with much higher shear thinning and also larger viscosity (Table 2). This phenomenon is likely because of the strong floc formation inducing a gel-like structure as a result of APP addition, in line with the previous study.^41^

**Table 2:** Summary of Obtained Consistency Index, Flow Behavior Index, and Yield Stress of Different Pickering-HIPE-Based Inks^a^

samples	yield stress (Pa)	consistency index (Pa s^n^)	flow behavior index	*R*^2^
control	0.01 ± 0.001^a^	0.3 ± 0.01^a^	0.99 ± 0.001^f^	0.983
E-CNC1	0.53 ± 0.002^b^	1.0 ± 0.02^b^	0.86 ± 0.002^e^	0.991
E-CNC4	0.78 ± 0.003^c^	2.6 ± 0.03^c^	0.79 ± 0.003^d^	0.996
E-CNC1/AP	2.24 ± 0.012^d^	4.8 ± 0.05^d^	0.67 ± 0.002^c^	0.998
E-CNC2/AP	2.18 ± 0.014^d^	7.9 ± 0.04^e^	0.57 ± 0.003^b^	0.988
E-CNC3/AP	3.30 ± 0.011^e^	10.7 ± 0.05^f^	0.44 ± 0.001^a^	0.989
E-CNC4/AP	3.99 ± 0.013^f^	11.1 ± 0.04^g^	0.45 ± 0.002^a^	0.996

a ^a–g^Means (three replicates) within each column with different letters in the same column are significantly different (*p* < 0.05): Duncan’s test.

In Figure 7a, the shear stress of all Pickering-HIPEs was increased progressively as the shear rate increased, followed by a linear rise. As expected, the shear stress at a given shear rate was higher for CNC-containing Pickering-HIPEs with larger CNC amounts. The trend shape curves of shear stress versus shear rate for CNC-contained HIPEs formulated by APP were comparable to those with no added APP, yet with much higher values (Table 2). Accordingly, a Herschel–Bulkley model was also applied to fit curves of shear stress–shear rate, where a curve fitted with a dashed type is also included in Figure 7a. Thus, the fitted results of the consistency index (*k*), flow behavior index (*n*), yield stress (τ_0_), and correlation coefficient (*R*^2^) are also summarized in Table 2. It was detected that the shear stress–shear rate curves for Pickering-HIPEs were fitted well by Herschel–Bulkley. As expected, the *k* was increased with an increase in CNC content, which is well in accordance with the viscosity measurement results. The *n* value of CNC-containing Pickering-HIPEs with no added APP was less than 1, which notably reduced as the CNC content increased. This proposes that the shear-thinning property became more noticeable with increasing CNC concentration. This could be a sign of the easy breaking down of some local aggregates, which become less strong at the higher shearing forces, therefore offering some degree of shear thinning. The flocculated emulsions exhibit enhanced shear-thinning behavior and higher viscosity compared to the nonflocculated emulsion. This is attributed to the screening of some of the continuous phases within the aggregate structures, not being able to flow, which then leads to increased viscosity. Upon the application of shear stress, the breakdown of the flocculation structure results in a shear-thinning response, characterized by a decrease in viscosity. Consequently, E-CNC3/AP and E-CNC4/AP displayed significantly higher viscosity values, accompanied by increased droplet flocculation. As the aggregates are fully broken down at higher shear rates, Newtonian behavior is expected and is indeed observed (Figure 7a).

Table 2 also shows that CNC-containing Pickering-HIPEs including APP possessed a considerable decrease in the flow behavior index, presenting well-defined shear-thinning behavior. As Figure 5 illustrates, a high level of flocs was detected in the CNC-contained Pickering-HIPEs having APP, where their aggregated droplets are expected to be broken into smaller clusters under increasing shear, leading to a strong shear thinning. The τ_0_ values of Pickering-HIPEs are in the range of 0.43–3.99 Pa (Table 2). Again, the τ_0_ values increased with increasing CNC content, whereas the samples with APP showed a higher value of τ_0_. This yield stress enhancement of CNC-containing Pickering-HIPEs including APP may be attributed to the higher effective volume fraction of the flocculated structure, where a more developed gel structure could form in these samples.

#### 3.2.5 Amplitude and Frequency Sweeps of Pickering-HIPE-Based Inks

Figure 7b,c shows the oscillatory rheological parameters detected by amplitude (strain) and frequency sweep measurements, which can give valuable information regarding the impact of CNC and APP on the mechanical strength and the yielding of Pickering-HIPEs. Previously, printing HIPE-based inks with higher CNC content in the presence of APP produced a floc system with strong shear-thinning behavior, higher viscosity, and greater yield stress as revealed by the rheological experiments. Thus, it is possible that the HIPEs including APP more effectively improve the elastic elements of the viscoelastic property, possibly due to the promotion of bridging or depletion flocculation of oil droplets. All of the Pickering-HIPEs prepared by CNC or CNC/APP behaved as gel-like systems, where the elastic modulus, *G*′ (γ), was higher than the viscous modulus, *G*″ (γ), over the entire strain range utilized inside the linear viscoelastic region (*LVR*) (Figure 7b). Alternatively, the yielding properties and gel strength were notably affected by the CNC content or the presence of APP in the HIPEs. The viscoelastic moduli increased progressively with increasing CNC levels in the system with no added APP. Since the oil volume fraction in the emulsion was high (0.74), the Pickering-HIPEs could be supposed to be characterized by the contribution from the O/W interfaces. This offers an estimated impression regarding the basics of Pickering stability against coalescence, wherein the CNC surrounds the oil droplets and can promote a weakly structured emulsion. Compared to HIPEs solely stabilized by CNC, the elastic modulus in *LVR* (*G*′_LVR_) increased more than 10-fold for CNC-contained Pickering-HIPEs containing APP (Figure 7b), proposing a co-stabilization of CNC and APP on the consistency of the HIPEs. In this kind of system, the development of an elastic behavior is most likely achieved by the formation of an emulsion gel (i.e., the large network of flocculated emulsion droplets), enhancing the particle network structuring. Indubitably, outside *LVR*, the absolute values of *G*′ (γ) and *G*″ (γ) need to be dealt with caution as the system response is no longer linear and the exact meaning of these moduli is less clear-cut. However, the fact that the viscoelastic moduli reduce is a sign of an altering (breakdown) of any stress-supporting structure/network in the system. All emulsion samples thus presented a yielding behavior at higher strain values, with the critical strain at the crossover or yield point (the point where *G*″ becomes larger than *G*′) ranging from 3.87 to 16.55.

To further analyze the response of the Pickering-HIPEs to the applied rate of deformation, a frequency sweep measurement was also performed (Figure 7c). In contrast to strain sweeps, more priority can be focused on exploring the tendencies and variations in the viscoelastic values, instead of the definite peaks (like strain overshoot) or transitions, once investigating the frequency scans.^47^ A low-frequency strain allows the materials sufficient time to relax, and therefore, the fluid-like flow features prevail. In this regard, with increasing frequency, the materials behave progressively in an elastic style, as the material has less time to relax for high-frequency deformations. Therefore, *G*′ rises with the increase in the frequency. The changes in *G*′ *with angular frequency* increase with viscoelastic compounds. With regard to the gel-like HIPEs, the stronger the gel strength, the lower the dependence of elastic modulus on frequency. Figure 7c shows the curves of *G*′ (ω) and *G*″ (ω) as a function of angular frequency, which indicate that the CNC-contained Pickering-HIPEs that include APP had a strong gel strength, with a trivial dependence of elastic modulus on the applied frequency. In these systems, *G*′ (ω) values were always higher than *G*″ (ω) over the entire measured frequency range. As suggested by the initial *G*′ values at low frequency, the addition of APP has a noticeable effect on the gel strength of the Pickering-HIPEs, which is according to the amplitude sweep results. It is worth mentioning that though a quasi-solid behavior was prevailing for E-CNC1 or E-CNC4, at the low frequencies (<1 Hz) (i.e., *G*′ > *G*″), there was a regional monotonic increase in moduli at higher frequencies. This led to *G*″ (ω) progressively getting closer to *G*′ (ω) until a crossover point (i.e., *G*′ = *G*″), highlighting the breakdown of the systems. This reveals the greater dependence of response on the frequency or in other words an inferior gel strength. Actually, these HIPEs with no added APP even offered a principally flow-dominated behavior (“liquid-like” feature) at low frequencies with overlapping values of *G*′ and *G*″ already at the lowest measured frequency.

#### 3.2.6 Time-Dependent Rheological Behavior of Inks

The suitability of HIPEs to develop printing inks also relates to their structural recovery. Hence, a five-interval thixotropy test (5-ITT) was conducted in a controlled rate mode, which can simulate the viscosity alteration of the emulsion-based inks over time before (low shear), during (high shear), and after (low shear) 3D printing (Figure 7d). With increasing shear rate from 0.1 to 100 s^–1^, a shear-sensitive behavior was detected in all HIPEs with a notable viscosity decline, which further confirms the *pseudoplastic* behaviors of the Pickering-HIPEs.^14^ A quick decrease in viscosity offered a breakdown of the structural arrangement of aggregate droplets within HIPEs, mimicking the time of emulsion passing through a narrow nozzle of a 3D printer. With readjusting the shear rate to an initial value (0.1 s^–1^), the structure of HIPEs could be partly reestablished rather quickly with a lower viscosity value compared to the first stage. This represents a capacity of HIPEs’ structural recovery, which simulates the emulsion sufficiently restoring its mechanical strength after passing through the 3D-printer nozzle to resist the repeated load of layers. Furthermore, the CNC-contained HIPEs with APP offered a strong thixotropic property whose viscosity showed a higher value compared to HIPEs solely stabilized by CNC. This highlights the ability of these samples to recover from a quick deformation, which can rearrange under shear, and a low-speed shear is then valuable to reorganize their structure, whereas the high shear can break their structure.

Another imperative factor in the ink flow behavior measurement is a creep and creep-recovery evaluation, which is beneficial to assess the performance of the dispersion with data from empirical approaches. The thixotropic feature of the inks was conducted by investigating the viscoelasticity of the Pickering emulsions through a creep and creep-recovery test. This experiment includes the utilization of constant applied shear stress; when the stress is removed, the viscoelastic compound displays a recovery to its initial shape or a progressive reduction in deformation over time. To evaluate the oscillatory yield stress (*G*′ (τ) = *G*″ (τ)), first, the viscoelastic evolution of Pickering-HIPEs as a function of the applied oscillation stress was assessed (Figure 7e). As expected, at a low shear rate (<1 Pa), *G*′ (τ) was higher than *G*″ (τ) for all HIPEs, which endow a characteristic gel-like structure. In this case, the viscoelastic moduli were moved to a higher value by increasing the CNC ratio in the presence of APP, which denoted that the systems were strengthened. As also visualized, the extent of *LVR*, i.e., the stress range inside which no structure breakdown happens, became larger after the addition of higher CNC content with APP. At a certain stress point, the dynamic moduli possessed a crossover, where the samples displayed a prevailing gel-like behavior below the crossover stress (*G*′ > *G*″) and more of a viscous-like property above it (*G*″ > *G*′). This presents an intersection of viscoelastic parameters, which can serve as the oscillatory yield stress. The stress sweep data also showed that the oscillatory rheology could monitor the changes in the yield stress of Pickering-HIPEs, which offered a decent approximation for static yield stress measured to those obtained through fitting of the Herschel–Bulkley model to stress–shear rate data (Table 2).

As a recoverable strain, creep compliance (*J*(*t*)) was detected by a maximum point of deformation before removing the load and the extent of deformation after the recovery period. The maximum *J*(*t*) and relative recovery parameters can be obtained from the creep-recovery curve. As Figure 7f illustrates, the magnitude of creep compliance regarding Pickering-HIPEs solely stabilized by CNC was 30- and 56-fold lower compared to CNC-contained Pickering-HIPEs including APP, signifying stronger solid-like characters of the latter samples. These results agree well with those measured by the oscillatory assay. The recovery phase of the creep evaluation can be considered as the extent of reducing ink deformation upon stress removal, where a greater relative recovery relates to higher elasticity and a gel-like structure. The addition of APP to the CNC-containing HIPEs enhanced the relative recovery properties. This denotes more elasticity with a strong stable structure with a lower relative recovery, which proposes the development of a strengthened ink structure induced by the addition of APP. The creep-recovery measurement proposed that a reversible network matrix was formed in the CNC-containing Pickering-HIPEs containing APP with the restoration of the original structures after the breakdown. To sum up, the results detected by creep-recovery measurements showed that the introduction of APP in the Pickering-HIPEs strongly affected the deformation recovery of elastic and viscous components regarding viscoelastic characters in the system, whose outcomes strongly relate to the data of discussed time-dependent 5-ITT.

#### 3.2.7 Lissajous Plots Analysis

**Figure 8:**
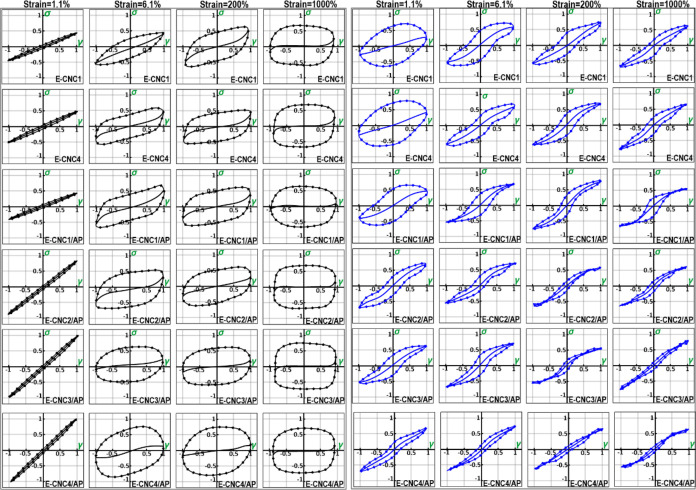
“Elastic” (black) and “viscous” (blue) Lissajous–Bowditch plots as a function of amplitude (a frequency of 1 rad s^–1^). Stress and strain results are normalized with a maximum stress/strain in the oscillation cycle.

An insight into the structural breakdown or deformation of Pickering-HIPEs during large strains can yield informative data on highly nonlinear rheological behavior by elastic and viscous Lissajous curves. Commonly, the Lissajous–Bowditch plots measured with LAOS represent a rapid evaluation of the structural progress within an actual physical material processing such as 3D printing, accounting for the microstructure breakdown of HIPES upon large deformations. The elastic (Figure 8, left) and viscous (Figure 8, right) Lissajous plots of Pickering-HIPEs monitor the response change at different strains. The dotted closed lines show the whole stress, although the solid lines present the elastic or viscous stress within the elastic and viscous Lissajous plots, respectively. All of the Pickering-HIPEs illustrated the elliptical Lissajous loop at a strain of 1.1%, signifying a linear viscoelastic property. The magnitude of distortion increased, and the ellipse-like shape changed to a parallelogram-like shape when strain increased. Such behavior shows a highly nonlinear property because of an increased viscous dissipation. This leads to an evolution from elastic to viscous dominance, which signifies the flow, yielding, and recovery of Pickering-HIPEs. As reported, the shape distortion of the Lissajous curve can be elicited by different microstructural characteristics and magnitude of structural response to large deformations.^48^

By comparison of Pickering-HIPEs, it can be observed that the depletion-flocculated HIPEs (i.e., E-CNC3/AP and E-CNC4/AP) showed a greater enclosed area once they were exposed to a similar strain. This finding proposed that the CNC-contained Pickering-HIPEs with APP had an inferior elasticity and were more prone to considerable deformation. This result revealed that the depletion-flocculated HIPEs needed an increased dissipated energy to obtain the nonlinear responses. The obtained result is supportive of the outcome revealed by the creep-recovery measurements (Figure 7f). To summarize, the interfacial assembly approach demonstrates an impact on the durability and viscoelasticity of the prepared Pickering-HIPEs, especially that of contained APP, which exhibits a substantial role in contributing to the nonlinear viscoelastic behavior of the system. Figure 8 on the right shows the area of viscous Lissajous plots, whose enclosed area decreased with increasing strain. A reduction of dissipated energy illustrated by decreasing area showed the emergence of shear-thinning features in a high strain rate region.^48^ A lower viscosity is associated with a lower energy dissipation. This functionality may present HIPEs (especially E-CNC3/AP and E-CNC4/AP) with an opportunity to extrude from a narrow nozzle during the 3D-printing process under a suitable shearing force.^49^ At a strain amplitude of 200 or 1000%, a separate small secondary loop was found regarding E-CNC3/AP and E-CNC4/AP, showing the incidence of a plastic structural network and the consequence of microstructural rebuilding following recoverable structural failure. This is likely associated with the reality that the time scale for structural retrieval of transient elastic stress is quicker compared to newly elastic deformation accumulation. It was obviously noticed that CNC-containing Pickering-HIPEs with APP possessed a clear secondary loop, which could show a superior thixotropic restructuring time scale.

## 4 Characterization of Pickering-HIPE-Based Inks of 3D-Printed Objects

### 4.1 Scaffold Morphology

**Figure 9:**
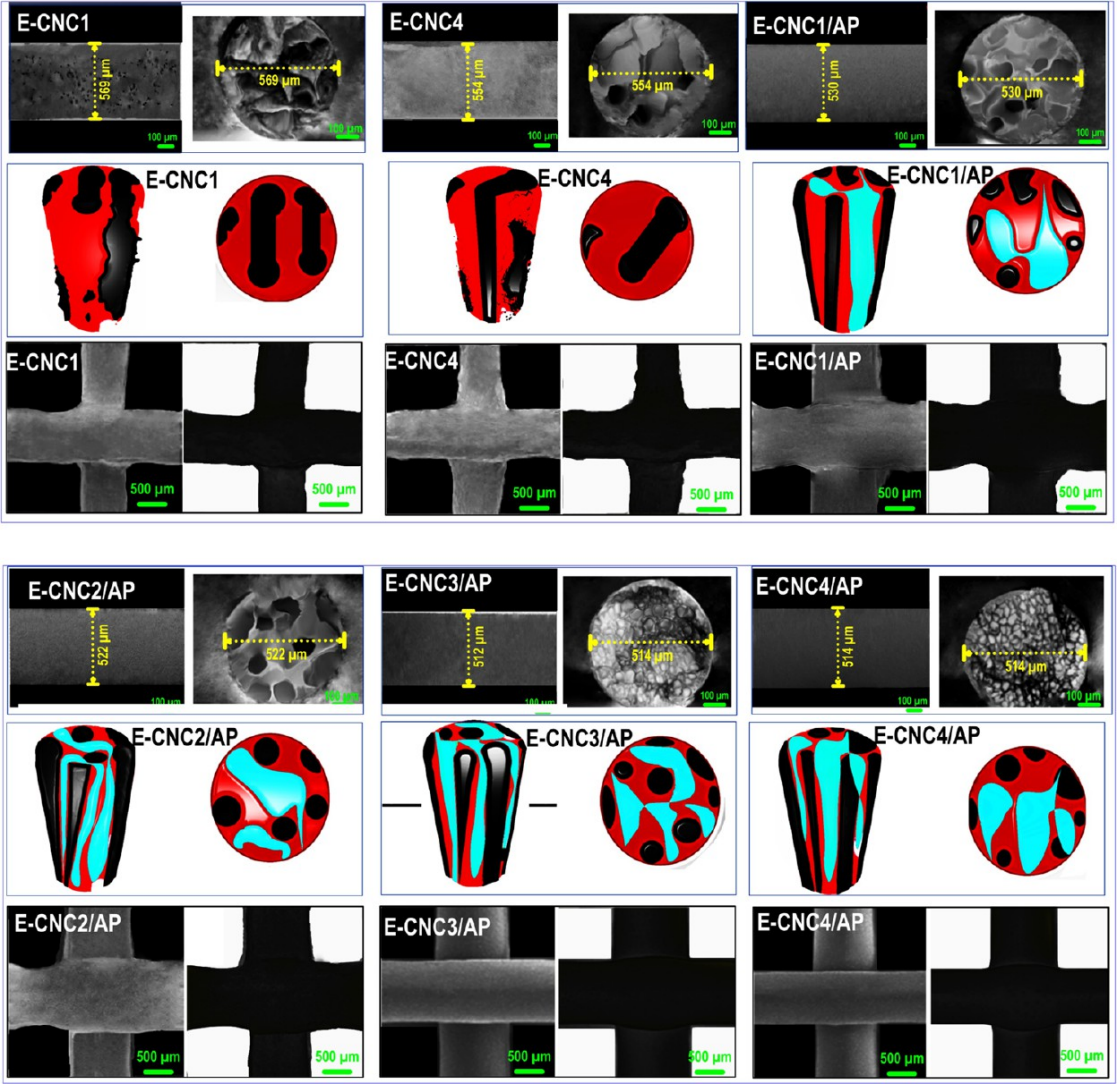
(Row *i* in each sample) SEM images of 1D filament, presenting their relevant photomicrographs of the surface (left) or cross-sectional rupture (right) of different printed filaments. (Row *ii* in each sample) Experimental results of segmented images obtained in a “thin” filament: (left) 3D and (right) *X*–*Y* cross section. (Row *iii*) FE-SEM images of the surface of 2D printed filaments (0–90° log-pile structure).

Until this point, the obtained morphological and rheological results demonstrated that a synergistic combination of CNC and APP promoted depletion flocculation in the HIPE-based inks (particularly E-CNC3/AP and E-CNC4/AP samples). This led to the formation of a gel-like structure, which can contribute to the creation of high-performance porous 3D-printed structures. The microstructure of the 1D filament evaluated by SEM is illustrated in Figure 9 (row *i* in each unit). The 1D filament printed by E-CNC1 and E-CNC4 inks possessed an irregular geometry with diameters of about 569 and 554 μm, respectively. Similarly, the cross section of the fracture surface of the filament produced by these inks was rough with several irregularities on the surfaces. In contrast, E-CNC1/AP, E-CNC2/AP, E-CNC3/AP, and E-CNC4/AP inks offered an even 1D filament surface with diameters of about 530, 522, 512, and 514 μm, respectively. In addition, the rupture surface of the 1D-like filament also offered a highly porous structure, which could lay the basis for the printing of superior, more intricate objects. Figure 9 (row *ii* in each unit) illustrates a typical cross-sectional image derived from the reconstructed 3D volume data and the corresponding segmentation results. When using conventional absorption-contrast imaging, segmentation can straightforwardly be achieved by setting the threshold values based on the contrast (i.e., absorption) of the reconstructed 3D data, as each material component exhibits distinct contrast levels. In contrast, phase-contrast imaging poses a more significant challenge for segmentation, as each material component displays similar contrast levels with the only notable contrast differences appearing at component boundaries, manifesting as black–white fringes. To segment the 3D volume images, we employed the “watershed segmentation” function within Fiji’s “Morphological Segmentation” plugin for the cracks, and a deep learning approach utilizing SegNet and MATLAB for the 1D filament. The results are depicted in Figure 9 (row *ii* in each unit), demonstrating the successful segmentation of the reconstructed 3D volume data using this procedure. Nevertheless, regions within the reconstructed images occasionally exhibit contrast levels between a crack (air) and a void, particularly when apparent voids are observed in advance of the crack tips.

Alternatively, the 2D printed filaments (a 0–90° log-pile structure) for “E-CNC1/AP”-“E-CNC4/AP” showed a fairly regular structure, which was geometrically ordered and interconnected as presented in Figure 9 (row *iii* in each unit). Once again, E-CNC1 and E-CNC4 presented some degree of shape deformation with curve-like angles, suggesting structural instability. This also led to some levels of extension of the line-edge roughness compared to a 3D structure printed with “E-CNC1/AP”-“E-CNC4/AP” inks. As mentioned earlier, the development of a gel-like structure in the highly depletion-flocculated HIPEs might describe this difference in precise geometry and shape fidelity.

**Figure 10:**
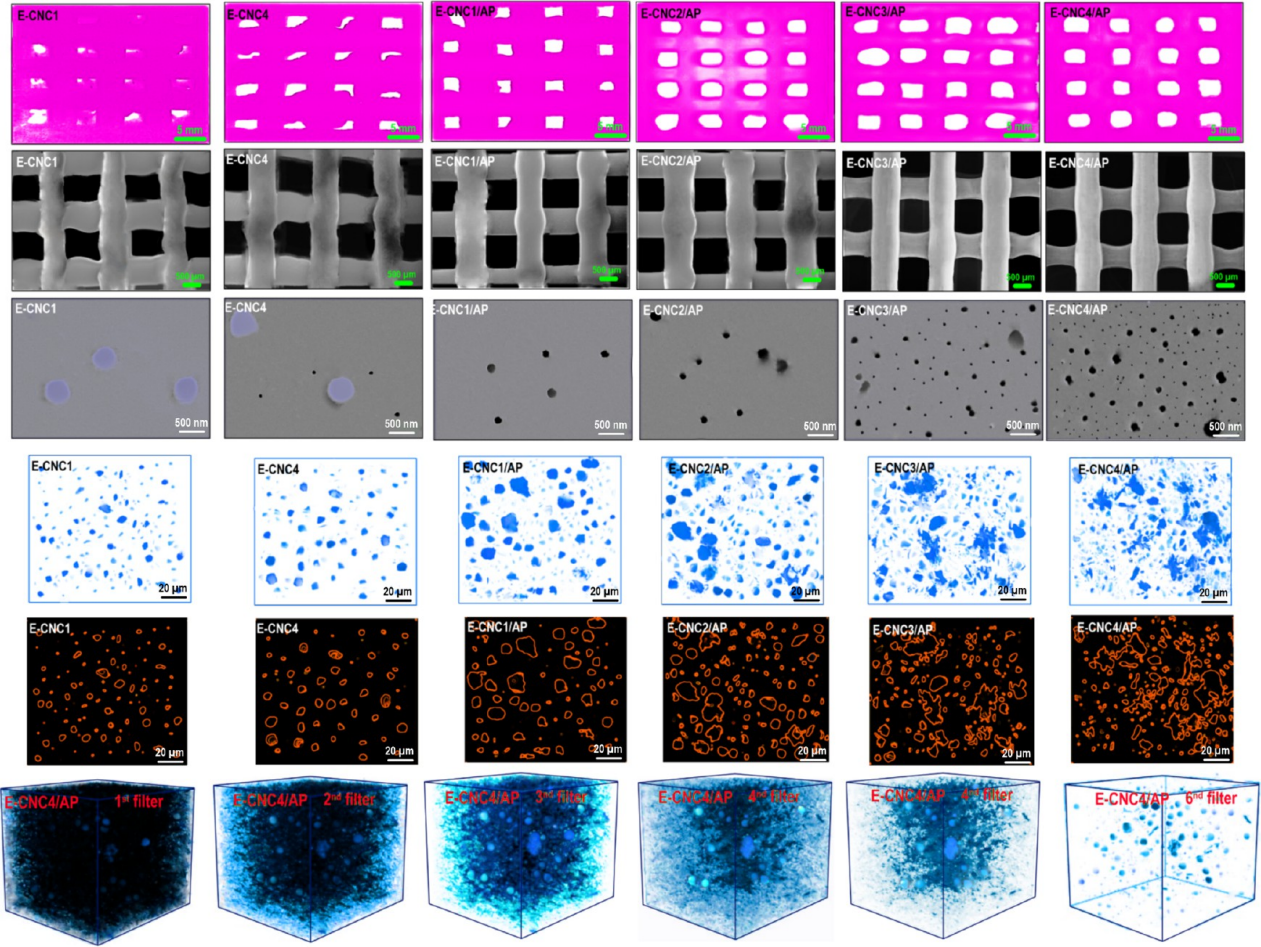
(Row *i*) Printing quality images of 3D-printed objects. (Row *ii*) SEM of 3D-printed-grid structures. (Row *iii*) FE-SEM images of the surface of freeze-dried 3D-printed objects. Extracted particle agglomerations seen in the *z*-axis using the FIJI software package (Row *iv*) with and (Row *v*) without lens correction. (Row *vi*) Example of the evolution of the 3D views by optimizing the threshold filter and extracting particle agglomerations.

Figure 10 (row *i*) illustrates the optical images of the 3D-printed objects. The morphology of 3D-printed E-CNC3/AP and E-CNC4/AP scaffolds showed a higher printing performance with precise geometry and enhanced shape fidelity. They displayed a typical open cell with a geometry with an ordered and interconnected structure. In this case, an improved elastic modulus with a higher thixotropic behavior of highly depletion-flocculated HIPEs led to better spatial resolution and printing performance. The printed structures based on E-CNC1 and E-CNC4 scaffolds, by contrast, had irregular geometries, with the printed parts and cells inside their matrix clearly seen to be nonuniform. As a result, these can present inferior printing quality and poor structural stability of 3D objects. Figure 10 (row *ii*) also shows the freeze-dried printed grids, presenting that E-CNC3/AP and E-CNC4/AP exhibited superior scaffold assembly with precise shape retention. In light of the viscoelastic properties (Figure 7b,c), thixotropic behaviors (Figure 7d,f), and nonlinear stress response (Figure 8) of the highly depletion-flocculated HIPEs, it was primarily established that E-CNC3/AP and E-CNC4/AP inks possessed excellent viscoelasticity, featuring nonlinear elastic characteristics that resulted in exceptional printing performance following 3D printing. The printability index (Pr) was also measured in freeze-dried printed grids. The Pr is a measure of the development of a perfect square geometry, ranging from 0 to 1, and is related to the printing pattern accuracy of an axial pore in the *XY* plane. A Pr value of 1 indicates a precise square shape, while values less than 1 indicate a round shape and values greater than 1 denote an irregular shape.^15^ According to the obtained quantification results, the Pr values for E-CNC1 and E-CNC4 were found to be 1.34 ± 0.08 and 1.33 ± 0.10, respectively, indicating a poor printing quality (Figure 10, row *ii*). In contrast, E-CNC3/AP and E-CNC4/AP possessed Pr values of 0.90 ± 0.09 and 0.99 ± 0.07, respectively, which reflect a high printing quality (Table S1). Notably, the pattern shape of E-CNC4/AP was only slightly rounded, with a Pr value close to 1. This suggests that the shape fidelities of E-CNC3/AP and E-CNC4/AP can be maintained even when additional layers are deposited onto their structure.^15^

Representative SEM images of the freeze-dried printed objects are also provided in Figure 10 (row *iii*). By analysis of SEM images, the microstructure of E-CNC1 was characterized to be a compact structure with some irregularity on its surface and no apparent pore structure within the matrix. E-CNC4 seemed also to be notably uneven, also having an uneven microstructure. Remarkably, printed E-CNC3/AP and E-CNC4/AP showed a 3D interconnected porous structure with a distribution of aperture diameters of nanometer size, ranging from about 80 to 600 nm. Compared to E-CNC3/AP, E-CNC4/AP presented a more randomly opened macroporous structure within an interconnected matrix possessing thicker pore walls. The resulting thick pore wall can endow the printed shape with a more supporting capacity and therefore may lead to enhanced adsorption. Thus, this meets the requirement for potential applications in drug delivery. Figure 10 (panels *iv*–*vi*) presents 3D views of 3D structures generated using FIJI software, illustrating the ascertained particle agglomerations using the scaffold model through progressive threshold filtering. Specifically, Figure 10 (panel *iv*) provides a 2D comparison of particle agglomeration in scaffolds having different particle contents. As can be observed, the level of agglomerations is slightly increased with the 3D structure formulations involving APP. Nevertheless, particle agglomeration in these highly depletion-flocculated HIPEs is high (Figure 10, panel *v*). This suggests that in 3D structures of E-CNC4/AP, the particles cannot be evenly dispersed in the matrix likely due to the depletion mechanism. That is to say, this agglomeration of the particles occurs, which may result in the formation of more compact structures (Figure 10, panel *vi*).

### 4.2 Mechanical Strength of 3D-Printed Constructs

**Figure 11:**
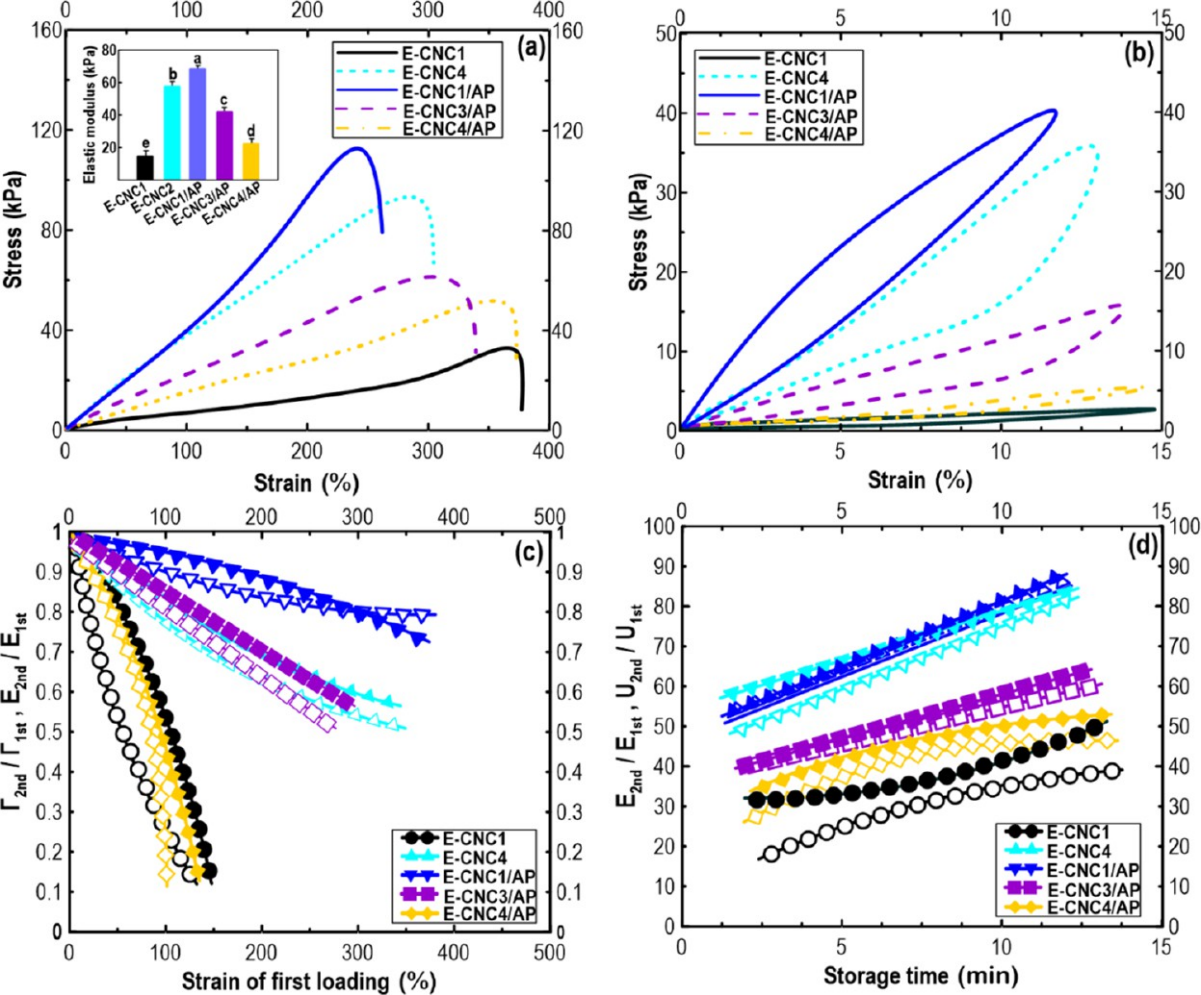
(a) Stress–strain curves of different 3D-printing architectures. The inset shows the means (three replicates) of elastic modulus, '*E*'. Data with different letters are significantly different (*p* < 0.05). (b) Curves of loading–unloading cycles. (c) *Γ*_second_ /*Γ*_first_ and *E*_second_/*E*_first_ as a function of the strain of first loading in different printed objects. (d) Proportions of elastic modulus (*E*) and energy dissipation (*U*) upon the second loading–unloading cycle to those during the first one for the relaxed and notched samples kept at 37 °C were plotted against different storage times.

The mechanical properties, including elastic modulus (*E*), fracture energy (Γ), and the underlying toughening mechanism, were assessed for the 3D-printed objects (Figure 11). The mechanical data revealed that the lowest elastic moduli (*E*) were detected to be 16 and 22 kPa for E-CNC1 and E-CNC4/AP, respectively. On the other hand, the maximum *E* values, 58 and 79 kPa, were observed for E-CNC4 and E-CNC1/AP (Figure 11a). This trend can be attributed to the fact that these latter samples exhibited a lower level of porosity compared to those of E-CNC3/AP and E-CNC4/AP, which can contribute to presenting a more mechanically rigid structure. Comparing E-CNC4 and E-CNC1/AP, it was found that 3D-printed CNC1/AP provided greater toughness, as illustrated by the larger area under the stress–strain curve. The greater area under the curve is a measure of a higher maximum energy dissipated during the fracturing of a material.^43^ The enhanced toughness in E-CNC1/AP can be credited to its low porosity level and its specific formulation, which contributed to its improved mechanical properties. These findings underscore the importance of optimizing the emulsion-based ink flocculation and 3D-printing parameters to achieve the desired mechanical performance in fabricated objects.

To gain a deeper understanding of the toughening phenomenon and fracture mechanism in 3D-printed structures, loading and unloading evaluations were conducted (Figure 11b). As demonstrated in the loading–unloading curve, below the yield strain of the printed samples (at a small tensile strain), E-CNC1/AP displayed a prominent hysteresis feature and maintained a significant level of persistent deformation upon unloading. Additionally, E-CNC4 also showed a higher degree of hysteresis, while other printed structures, especially E-CNC1 and E-CNC4/AP, did not exhibit any hysteresis behavior. This observation suggests that E-CNC4 and E-CNC1/AP possess better energy absorption capabilities, which contributes to their enhanced toughness.^15^ The hysteresis in these samples can be attributed to their viscoelastic nature and low porosity levels. This allows them to more easily dissipate energy during the loading and unloading process. The energy dissipation process helps to relax stress and delay the onset of the fracture process. In turn, this translates to an improvement in the overall mechanical performance of the 3D-printed materials.^16^

To evaluate the disruptive strength, a ratio of fracture energy (*Γ*_second_) and elastic modulus (*E*_second_) in the second loading–unloading phase was compared with their values in the first loading–unloading phase (Γ_first_ or *E*_first_). As depicted in Figure 11c, all samples, except E-CNC1 and E-CNC4/AP, demonstrated a sudden decrease in the *E*_second_/*E*_first_ or *Γ*_second_/*Γ*_first_ ratio with increasing strain during the first loading–unloading cycle. This indicates that the elasticity of the printed matrices decreased as a result of breaking and alteration of the structure as the extension level was increased. These disruptive strength results for E-CNC1 and E-CNC4/AP demonstrated higher viscoelasticity and structural strength, as these samples maintained the highest elastic modulus and enduring deformation upon unloading.

Furthermore, Figure 11d shows the recoverability of the notched 3D-printed structures, revealing that both E-CNC1 and E-CNC4/AP regained approximately 75% of their elastic modulus (*E*) and 60% of their energy dissipation (*U*). This indicates a highly recoverable structure. These findings align with the results from Section 3.2.6, which discuss the time-dependent 5-ITT and creep-recovery data. It has been shown that energy dissipation in a multicomponent emulsion system is positively related to reduced droplet size and the presence of bridging flocculation between oil droplets.^43^ In this instance, rapid reformation of the oil droplet network and restoration of the original architecture can be attributed to the enhanced mechanical properties and recoverability of the 3D-printed structures. This phenomenon is likely due to the reorganization of the oil droplets, which allows for the reestablishment of their original hierarchical structure and interfacial interactions, thereby restoring the material’s mechanical integrity and viscoelastic properties.

### 4.3 *In Vitro* Curcumin Release from Prepared 3D Porous Scaffolds

**Figure 12:**
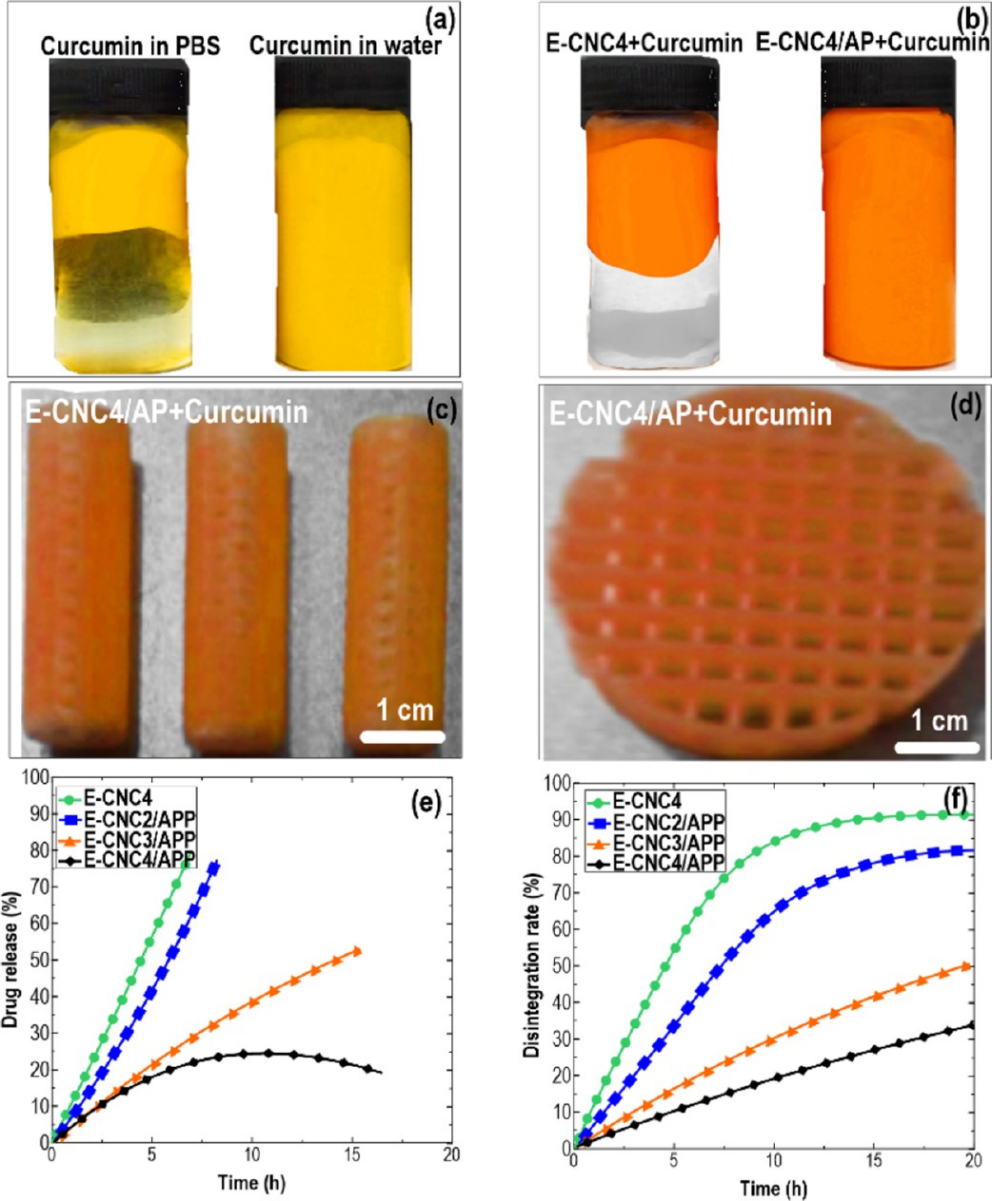
(a) Curcumin in water and PBS, (b) curcumin stabilization in E-CNC4/AP ink. 3D printing (c) capsules and (d) circular mesh structures. (e) Curcumin release plots from the E-CNC4/AP object. (f) Disintegration rate of E-CNC4/AP objects.

To evaluate the suitability of the developed 3D porous scaffolds as a platform for local drug delivery, curcumin was loaded into the as-prepared ink. Curcumin has received increased therapeutic attention because of its effectiveness in treating inflammatory and cancer diseases. In the current work, we dispersed hydrophobic curcumin into PBS or water, which, as expected, revealed poor solubility (Figure 12a). Yet curcumin was physically stabilized in highly depletion-flocculated E-CNC4/AP HIPE (Figure 12b). Once curcumin was incorporated into this “floc” HIPE (within oil droplets, in a much more hydrophobic environment) was entrapped and stabilized by the CNC/APP, which stay homogeneously distributed throughout the ink. Throughout our experimentation, we comprehensively evaluated the printable inks available, and owing to the exceptional printing performance of E-CNC4/AP, it was selected for the acquisition of the image depicting the dispersion of curcumin.

Thus, curcumin was added to E-CNC4/AP ink as this 3D-printed scaffold was found to be a highly porous structure with a distribution of pore diameters in the nanometer size range (Figure 10, Row *iii*). This ink also provided the highest elastic (Figure 7b) and thixotropic (Figure 7d,f) features. Thus, it was effectively used for the 3D printing of a capsule or circular mesh scaffold (Figure 12c,d), allowing the printed objects to retain their 3D shapes after printing. It was also shown that curcumin was homogeneously integrated and stabilized throughout the E-CNC4/AP ink. As can be seen, the introduction of curcumin had no impact on the 3D functionality of E-CNC4/AP during the printing process, offering the same excellent printing performance, printing precision, and toughness of the printing architectures as those of inks without the incorporation of the drug. Finally, the printed capsules or circular mesh scaffolds were freeze-dried.

All freeze-dried 3D-printed scaffolds (3D capsule and circular mesh) were assessed for their suitability as drug delivery systems under *in vitro* conditions (PBS, 37 °C) for 24 h. The released curcumin amount was evaluated through UV–vis spectroscopy. As Figure 12e illustrates, the E-CNC3/AP and E-CNC4/AP scaffolds offered the shortest times for total curcumin release, which were measured to be about 7.5 and 5 h. Curcumin release in the E-CNC4 scaffold was detected for 24 h, and data demonstrated that only half of the total amount of curcumin was released during that time. Because of its low solubility in PBS, the curcumin release is likely triggered by water ingress into the 3D-printed scaffold having a high porosity level, and the resultant progressive disintegration. Therefore, the releasing behavior of curcumin inside the oil droplets in highly depletion-flocculated HIPE inks can be related to the porosity level of 3D structures and their progressive disintegration. Accordingly, the disintegration of the 3D scaffold was also concurrently detected (Figure 12f) by collecting the residual inks at predetermined times (data not shown). Again, concerning curcumin release plots, E-CNC3/AP and E-CNC4/AP scaffolds offered faster disintegration of the 3D-printed scaffold compared to other 3D structures. The depletion effects and stabilization induced by the presence of APP in CNC-based HIPEs allow tailoring of the 3D-printed inks with improved printing quality and mechanical properties. These functionalities were reflected on the 3D objects printed from depletion-flocculated HIPE-based inks, presenting a higher degree of porosity with enhanced releasing behavior. This gives rise to a quicker water ingress into the scaffold and, therefore, a greater disintegration rate. As a result, the release of curcumin and disintegration of the 3D-printed E-CNC4/AP scaffold were higher than those of other samples.

## 5 Conclusions

Herein, we study the possibility of APP particles altering the colloidal stability of CNC-based HIPEs via the depletion-flocculation phenomenon so as to produce mechanically robust ink with improved printing quality and high porosity of 3D-printed objects. Such inks can be particularly useful in the delivery of bioactive components. We present a simple and sustainable method that utilizes CNC particles as an interfacial stabilizer of Pickering emulsions. This was combined with a nonadsorbing APP particle that induces tunable physical stability. Adding such sustainable particle promoted depletion flocculation of droplets with tailored flow behavior of Pickering-HIPEs, whilst higher CNC concentrations led to the formation of a gel-like structure. Our experimental data revealed that the depletion-flocculation effects as induced by the presence of APP in CNC-based HIPEs, while still retaining the stability of droplets against coalescence, offered tunable viscoelasticity, shear-thinning features, and thixotropic recovery. These properties provide a promising functional printing ink, having the appropriate rheological features for advanced 3D printing. The 3D-printed architectures printed by depletion-flocculated HIPEs were demonstrated to have brilliant printing performance and shape fidelity, which contributed to forming a materially tough 3D structure with a high maximum fracture energy. In this case, the freeze-dried 3D-printed scaffolds produced by depletion-flocculated HIPE developed a highly porous structure, where water simply diffused into their structures because of the existence of open pores in the nanometer size range. This can then trigger scaffold disintegration and curcumin release. Again, this 3D structure supported a higher drug release because of the increased porosity of 3D scaffolds, which finally involved a quicker disintegration rate. This variation in the release kinetics of the bioactive compounds could serve as a way to custom-design the drug delivery system, which is adapted to be consistent with the requirements of personalized drug delivery. Increasing effort and attention should be paid to the combination of multisystem mechanically robust emulsions with extrusion-based 3D printing in a more suitable and advanced fashion. As shown here, this has the potential to lead to preferred 3D-printing emulsion-based ink formulations, allowing for a higher shape fidelity and wider applicability in the future.

## Supporting Information Available

Physical stability of Pickering-HIPEs as affected by different levels of CNC and APP (Section S1); quartz crystal microbalance with dissipation monitoring (QCM-D) (Section S2); printability index (*Pr*) and filament diameter (Section S3); preparation of HIPEs as affected by oil fraction phase (Section S4); microstructure of HIPEs (Section S5); apparent viscosity (Section S6); strain and frequency sweeps (Section S7); and printing performance as affected by oil contents (Section S8) (PDF)
