# Supplementary material for: Depletion Flocculation of High Internal Phase Pickering Emulsion Inks: A Colloidal Engineering Approach to Develop 3D Printed Porous Scaffolds with Tunable Bioactive Delivery
Source: ACS Appl Mater Interfaces. 2024 Aug 7;16(33):43430–50. doi: 10.1021/acsami.4c11035 (PMC11345728; doi:10.1021/acsami.4c11035)
Supplement: Supplementary file 1 — am4c11035_si_001.pdf [file am4c11035_si_001.pdf]

<u>Supporting Information</u>:

Depletion flocculation of high internal phase Pickering emulsions: A colloidal engineering approach to develop porous scaffolds with tunable bioactive delivery

Mahdiyar Shahbazi[a] Henry Jäger[a]*, Delphine Huc-Mathis[b], Peyman Asghartabar Kashi[c], Rammile Ettelaie[d], Anwesha Sarkar[d], Jianshe Chen[e]*

[a]Institute of Food Technology, University of Natural Resources and Life Sciences (BOKU), Muthgasse 18, 1190, Vienna, Austria.

[b]*Université Paris-Saclay, INRAE, AgroParisTech, UMR SayFood, 91300 Massy, France*

[c]Faculty of Biosystem, College of Agricultural and Natural Resources, Tehran University, 31587-77871 Karaj, Iran

[d]Food Colloids and Bioprocessing Group, School of Food Science and Nutrition, University of Leeds, Leeds, LS2 9JT, U.K.

[e]Food Oral Processing Laboratory, School of Food Science & Biotechnology, Zhejiang Gongshang University, Hangzhou 310018, China

Corresponding Authors

Mahdiyar Shahbazi - University of Natural Resources and Life Sciences (BOKU), Muthgasse 18, 1190Vienna, Austria; Orcidhttps://orcid.org/0000-0002-2485-9130; Email: mahdiyar.shahbazi@boku.ac.at shahbazim00@yahoo.com

Henry Jäger - Institute of Food Technology, University of Natural Resources and Life Sciences (BOKU), Muthgasse 18, 1190Vienna, Austria; Email: henry.jaeger@boku.ac.at

S1. Physical stability of Pickering-HIPEs as affected by different levels of CNC and APP

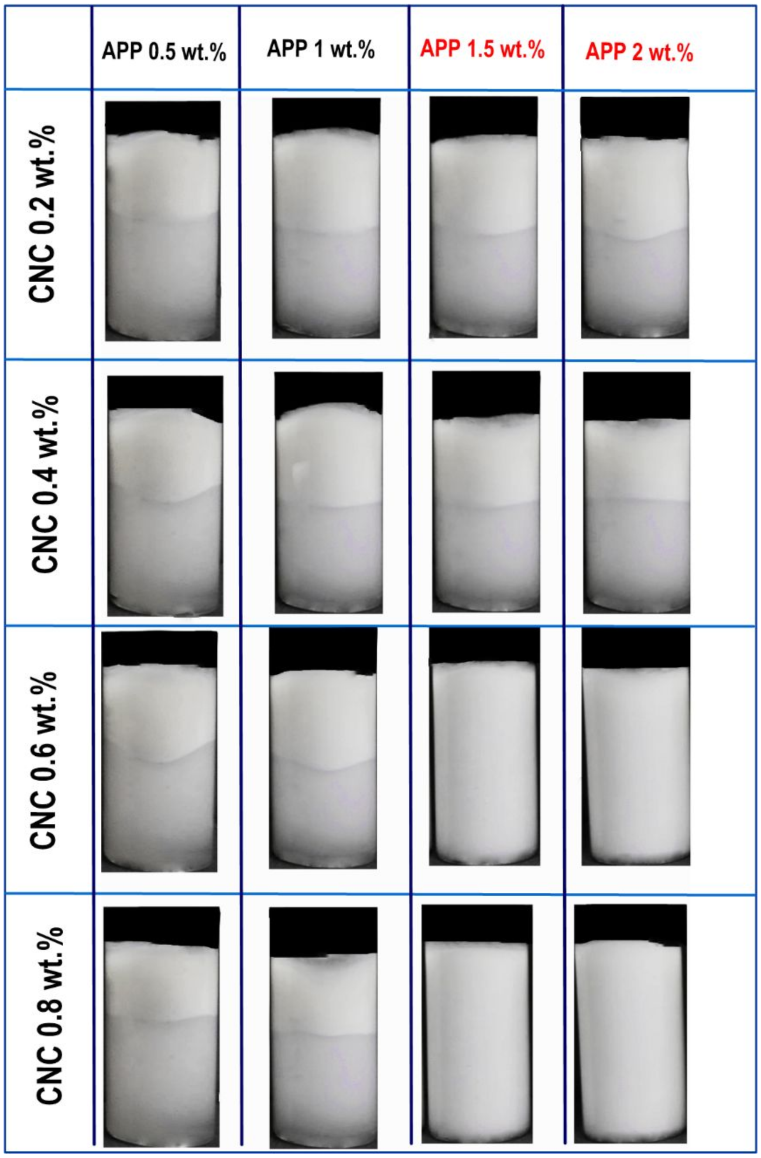

Figure S1. Physical stability of Pickering-HIPEs as affected by different levels of CNC and APP.

S2. Quartz crystal microbalance with dissipation monitoring (QCM-D)

A QCM-D (Q-Sense E4 AB, Gothenburg, Sweden) was used to evaluate the adsorption properties of Pickering-HIPEs with the ability to detect variations of resonance frequency (Δf) and energy dissipation (ΔD) of QCM-D sensors. The gold sensors with a fundamental resonance frequency of 4.95 MHz (QSX 301) were cleaned and coated with a monolayer of sunflower oil by spin coating at 2000 rpm for 2 min with the oil

solution (0.1 wt.% in chloroform). The experiment was conducted at a flow rate of 50 μL min^{-1} at ambient conditions, and the Milli-Q water was used to establish a signal baseline. The Sauerbrey equation was used to make a relationship between Δf and the coupled mass (Δm):

$$\Delta \mathrm{m} = -C\,({}^{\Delta f}\!/_{n}) \qquad \text{(S1)}$$

In the above equation, C is a mass constant depending on the physical property of the sensor (17.7 ng cm^{-2} Hz^{-1} for a 5 MHz crystal), and n is the resonance number of the sensor (n = 1, 3, 5, 9, 11, 13). For the sake of simplicity, only one harmonic (n = 5) was presented in the results here. In the current work, the fifth harmonic was employed to measure the dissipation shifts and frequency, as well as to obtain the corresponding mass change.

S3. Printability index (Pr) and filament diameter

Table S1. Summary of obtained filament diameter and printability index of different 3D printed samples.

Samples	Filament diameter (μm)	Printability index (*Pr*)
**E-CNC1**	569 ± 5.88^{e}	1.34 ± 0.08^{e}
**E-CNC4**	554 ± 4.04^{d}	1.33 ± 0.1^{e}
**E-CNC1/AP**	530 ± 3.65^{c}	1.11 ± 0.02^{d}
**E-CNC2/AP**	522 ± 2.79^{b}	1.05 ± 0.01^{c}
**E-CNC3/AP**	512 ± 2.86^{a}	0.90 ± 0.09^{a}
**E-CNC4/AP**	514 ± 3.42^{a}	0.99 ± 0.07^{b}

$^{a\text{–}e}$ Means (three replicates) within each column with different letters are significantly different ($p < 0.05$), Duncan's test.

S4. Preparation of HIPEs as affected by oil fraction phase

Pickering emulsions with varying oil fractions were prepared following the same procedure as the oil-in-water emulsions, with the only difference being the adjustment in oil fractions. In this case, the CNC and APP concentration was set at 0.8 wt.% CNC and 1.5 wt.% APP and the oil volume fractions considered were 65%, 80%, and 95%.

S5. Microstructure of HIPEs

Microstructures of emulsions were observed using an optical microscope (Leica DMD 108, Leica Microsystems Inc., Heidelberg, Germany) equipped with a camera. The emulsions were diluted by the continuous phase. A drop of diluted emulsion sample was deposited on the microscope slide and covered with a cover slip. All the measurements were performed at 25 °C.

Optical microscopy images of HIPEs revealed that the oil droplets were closely packed together and that they were dispersed throughout the aqueous phase (Figure S2, row *i*). The large oil droplets in some of these emulsions had polyhedral shapes, which can be attributed to their low Laplace pressure and close packing. When the oil concentration increased from 65% to 80 and 95% in these emulsions, the oil droplets were observed to become more densely packed in microscopy images. Moreover, as the oil concentration reached 95%, the size of droplets was increased. This occurred because the protein content, which helps cover the droplet surfaces, was insufficient to maintain stability at this higher oil concentration level. This observation is crucial for understanding the behavior of emulsions under varying oil concentrations and the role of proteins in maintaining their stability.

The impact of φ on HIPE formation was detected and images are illustrated in Figure S2, row *ii*. Using the inverted-tube method, gel-like emulsions were formed within the φ range of 65, 80, and 95%. With φ at 95%, however, the resulting emulsion was slightly flowable and unstable after 24 h storage. In the main body of the manuscript (Figure 4iv, Right), the HIPE with φ of 74% also held their weight once the tubes were inverted, suggesting that this system was structured with a gel-like behavior. As the optimal φ threshold for HIPEs was greater than 74%, our findings suggested that physically stable HIPEs could be produced at a CNC:APP ratio of >0.6:1.5 (wt.%) and a 65-80% (v/v) φ.

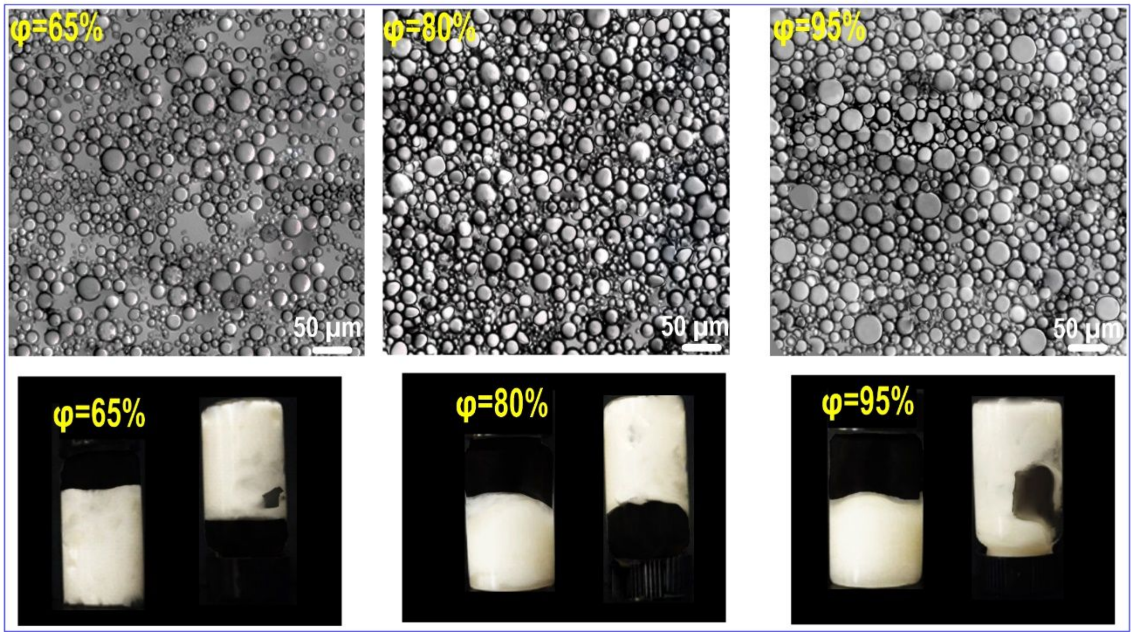

Figure S2. Optical microscopy images and visual observation of Pickering HIPEs (0.8 wt.% CNC and 1.5 wt.% APP) as affected by the content of the oil internal phase.

S6. Apparent viscosity

Examining the rheological properties of edible ink is crucial for its utilization in 3D printing applications. To ensure optimal performance, the ink must possess suitable fluidity for passage through the nozzle, rapid solidification post-deposition onto the platform, and sustained structural integrity over time. Consequently, diverse rheological assessment techniques were employed to evaluate the characteristics of HIPES in this context.

To achieve optimal functionality in 3D printing, the apparent viscosity of the ink should strike a balance: being low enough to facilitate smooth extrusion through the nozzle yet sufficiently high to prevent rapid spreading post-deposition. Figure S3a illustrates the variation in apparent viscosity with the shear rate for HIPEs containing diverse oil proportions. For all HIPEs, the data, when plotted on a log-log scale, exhibited an almost linear decline in the apparent shear viscosity with increasing shear rate (Figure S3a). The observed shear-thinning behavior in HIPEs can be attributed to the disruption of their network structure under the influence of shear forces. This characteristic is generally advantageous for edible inks, as it allows for easier extrusion through the nozzle without compromising structural integrity upon deposition. Shear-thinning inks facilitate smoother 3D printing processes by accommodating varying flow requirements during extrusion and post-deposition stages.

In the context of HIPEs, the viscosity tends to increase with oil content, as demonstrated in Figure S3a. This rise in viscosity can be attributed to the increased oil content causing oil droplets to be more densely packed together. As a result, a higher force is needed to induce flow, leading to an initial shear viscosity that escalates from approximately 1240 to 2010 Pa s when the oil content rises from 75% to 80% (Figure S3a). This phenomenon highlights the influence of oil content on the overall viscosity and flow behavior of HIPEs.

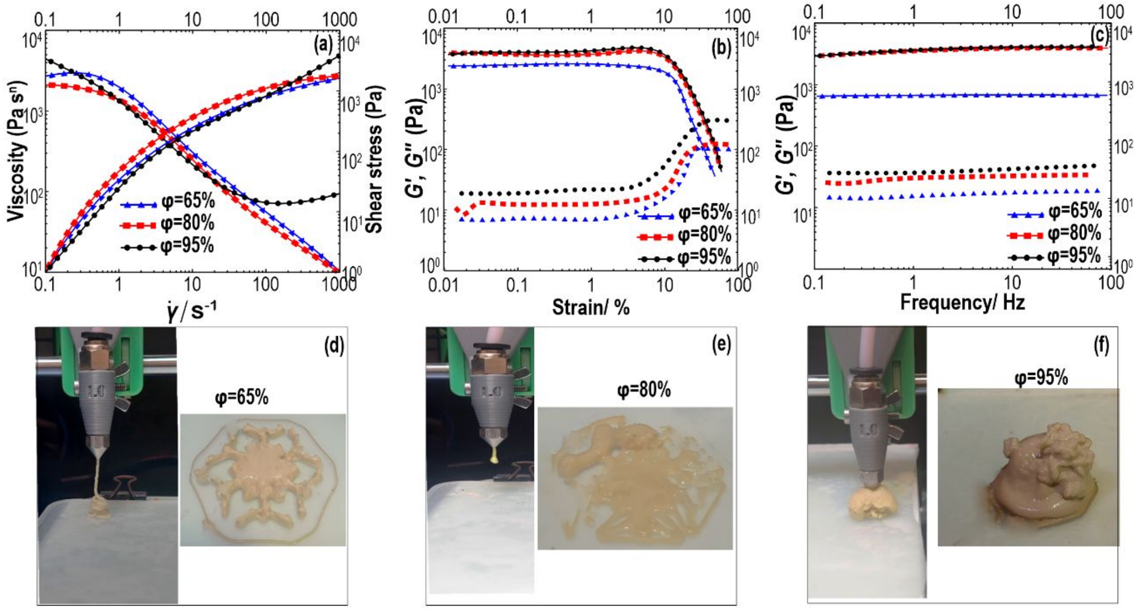

Figure S3. (a) The flow curve presents changes in the shear stress and viscosity with the shear rate. (b) Strain and (c) frequency sweeps, where G' is denoted by solid symbols and G" is denoted by open symbols. Printing performance of samples with (d) 65%, (e) 80%, and (f) 95% oil content.

S7. Strain and frequency sweeps

Strain sweep analysis showed that both G' and G'' remained relatively stable at low strains, suggesting a well-maintained network structure (Figure S3b). However, as the strain increased, these values decreased sharply. Within the LVR (a strain of lower than 10%), G' consistently surpassed G'', demonstrating that HIPEs exhibited predominantly elastic-like properties under these conditions. Beyond the LVR, G' diminished, while G'' increased with rising strain, signifying a transition from more elastic behavior to a viscous-like response. In summary, the G' and G'' values of HIPEs typically increased with rising oil content, which can be linked to the reasons discussed earlier for the increase in viscosity. This enhancement in both moduli suggests a more robust network structure and improved mechanical properties. To ensure accurate results and maintain the elastic-like behavior of HIPEs, the rest of the oscillation tests were conducted at a constant strain of 0.5%.

The change in shear modulus with increasing frequency was also measured for HIPEs with different oils (Figure S3c). G' was greater than G'' across the entire angular frequency range used, indicating that they remained solid-like at all frequencies. Both G' and G'' increased with increasing oil, which is consistent with the results discussed earlier.

S8. Printing performance as affected by oil contents

Printing performance can be assessed in terms of the resolution of deposited layers and shape fidelity, which is strongly associated with the flow behavior of printable inks and self-supporting features of 3D printed structures. To underline the versatility of fabricated 3D-printed starches, we evaluated the printing performance through the development of 3D-printed constructs. The printing quality images of 3D printing of a snowflake-shaped structure are also shown in Figure S3, Bottom. Following 3D printing, the HIPE with 65% (v/v) φ was successfully squeezed out from the nozzle tip (Figure S3d). However, HIPE with an 80% (v/v) φ could not be printed well, as its filament extruded non-continuously or extruded filament was interrupted. This is reflected in its 3D printing quality image, whose 3D structures showed an uneven shape, which spread over the surface (Figure S3e). This is likely due to the high viscosity value of HIPE with φ of 80%, where the pump pressure failed to provide sufficient energy to extrude the filament out of the nozzle tip. Furthermore, it should be noted that the 3D printing of HIPE with φ of 95% was also not successful as the nozzle tip was clogged during the 3D printing process (Figure S3f). These results were also consistent with the fact that emulsions at higher φ showed an increase in viscosity due to enhanced droplet packing.

S9. Printing performance as affected by both oil contents and CNC/APP ratio

In this series of experiments, the printing quality of HIPEs as influenced by oil contents and the addition of CNC/APP was assessed (Figure S4). In the sample containing 65% oil content, we successfully printed "star" shapes using HIPE inks. Post-printing, these shapes maintained their structural integrity, exhibiting no collapse or missing parts from the original design. Moreover, with increasing CNC level at a constant APP level the system was found to have a positive

influence on the printing accuracy. These findings might be attributed to the enhanced structural strength of relevant inks, which could contribute to the formation of a robust gel-like network. This improved structural integrity may help maintain the stability of the HIPEs even at higher oil droplet concentrations, preventing the issues observed in the rougher surfaces and demarcation lines.

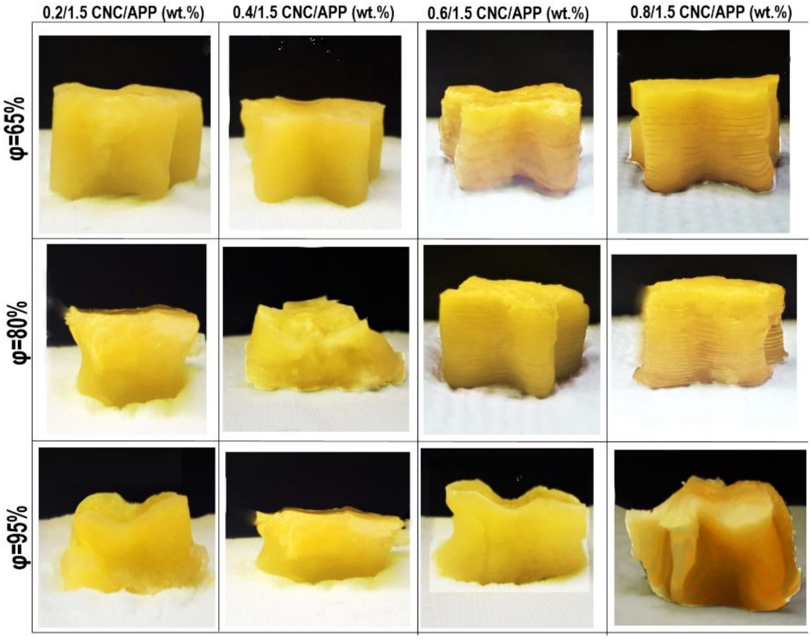

Figure S4. Printing performance as affected by both oil contents and CNC/APP ratio.

As the oil content increased in the HIPEs, the surfaces of the star-shaped prints deformed in time because of sagging, henceforth showed weak resolution and poor shape-fidelity (Figure S4). This phenomenon was primarily attributed to a decrease in HIPEs' stability especially in HIPEs with φ of 95%. As previously mentioned, the oil droplets tended to enlarge and exhibit less uniformity as the oil content increased to 95%, leading to less stable emulsions prone to separation. Consequently, the extruded filaments fractured during the extrusion process and failed to connect properly with the subsequent layer (data not shown).

It should be noted that, in the HIPEs with an 80% (v/v) φ, when 0.6 and 0.8 CNC wt.% were added in the presence of 1.5% APP, 3D printed structures showed better printing quality. This effect can be credited to the rise in viscosity, gel strength, and stability of their relevant inks. These enhancements allowed the inks to better retain their shape after printing, thus improving their overall performance and stability.
